# Hierarchical Individual Naturalistic Functional Brain Networks with Group Consistency Uncovered by a Two-Stage NAS-Volumetric Sparse DBN Framework

**DOI:** 10.1523/ENEURO.0200-22.2022

**Published:** 2022-09-07

**Authors:** Shuhan Xu, Yudan Ren, Zeyang Tao, Limei Song, Xiaowei He

**Affiliations:** School of Information Science and Technology, Northwest University, Xi’an, China, 710129

**Keywords:** naturalistic fMRI, neural architecture search, deep belief network, hierarchical functional brain networks

## Abstract

The functional magnetic resonance imaging under naturalistic paradigm (NfMRI) showed great advantages in identifying complex and interactive functional brain networks (FBNs) because of its dynamics and multimodal information. In recent years, various deep learning models, such as deep convolutional autoencoder (DCAE), deep belief network (DBN), and volumetric sparse DBN (vsDBN), can obtain hierarchical FBNs and temporal features from fMRI data. Among them, the vsDBN model revealed a good capability in identifying hierarchical FBNs by modeling fMRI volume images. However, because of the high dimensionality of fMRI volumes and the diverse training parameters of deep learning methods, especially the network architecture that is the most critical parameter for uncovering the hierarchical organization of human brain function, researchers still face challenges in designing an appropriate deep learning framework with automatic network architecture optimization to model volumetric NfMRI. In addition, most of the existing deep learning models ignore the group-wise consistency and intersubject variation properties embedded in NfMRI volumes. To solve these problems, we proposed a two-stage neural architecture search (NAS) and vsDBN model (two-stage NAS-vsDBN model) to identify the hierarchical human brain spatiotemporal features possessing both group consistency and individual uniqueness under naturalistic condition. Moreover, our model defined reliable network structure for modeling volumetric NfMRI data via NAS framework, and the group-level and individual-level FBNs and associated temporal features exhibited great consistency. In general, our method well identified the hierarchical temporal and spatial features of the brain function and revealed the crucial properties of neural processes under natural viewing condition.

## Significance Statement

In this paper, we proposed and applied a novel analytical strategy, a two-stage neural architecture search (NAS)-volumetric sparse deep belief network (vsDBN) model to identify both group-level and individual-level spatiotemporal features at multiscales from volumetric functional magnetic resonance imaging under naturalistic paradigm (NfMRI) data. The proposed particle swarm optimization (PSO)-based NAS framework can find optimal neural structure for both group-wise and individual-level vsDBN models. Furthermore, with well-established correspondence between two stages of vsDBN models, our model can effectively detect group-level functional brain networks (FBNs) that reveal the consistency in neural processes across subjects and individual-level FBNs that maintain the subject-specific variability, verifying the inherent property of brain function under naturalistic condition.

## Introduction

Recently, functional magnetic resonance imaging (fMRI) has been considered as an effective tool to explore functional brain networks (FBNs) and temporal responses (Damascelli et al., 2021). Previously, various studies rely on task-based fMRI (tb-fMRI; Cui et al., 2019) and resting-state fMRI (rs-fMRI; Catalino et al., 2020). However, the simplified stimuli adopted in task paradigms rarely occur in isolation in real life, thus it is unclear whether it can reveal the complex neural responses evoked in daily life (Hasson et al., 2010). Moreover, subjects struggle to maintain vigilance during resting-state scanning, thus microsleeps and head movements occur commonly (Vanderwal et al., 2015). To avoid the limitations of these paradigms, researches propose naturalistic paradigm, which employs rich multimodal and dynamic stimuli that resemble the perceptual and cognitive experiences in real life (Sonkusare et al., 2019), and offers a way to identify hierarchical spatiotemporal patterns.

Therefore, researches have employed NfMRI to examine FBNs using diverse computational methods, including general linear model (GLM; Beckmann et al., 2003), independent component analysis (ICA; Salman et al., 2019), and sparse dictionary learning (SDL; Ren et al., 2017a). Although these methods can detect meaningful FBNs, they ignore the hierarchical structure of FBNs because of their shadow nature (Ferrarini et al., 2009). To address the limitations of shallow models, increasing deep learning approaches have been proposed to construct hierarchical spatiotemporal features from fMRI data, including deep convolutional autoencoder (DCAE; H Huang et al., 2018), deep belief network (DBN; ZA Huang et al., 2021), and recurrent neural network (RNN; Cui et al., 2019). These models reveal great capacity in extracting hierarchical FBNs and temporal features from fMRI time series. However, fMRI time series can cause more intersubject variability across subjects compared with spatial volumes, which can affect the reliability of derived FBNs (W. Zhang et al., 2019; Y. Zhang et al., 2019). Therefore, whether/how to uncover the complicated hierarchical temporal and spatial organization of brain function from NfMRI volumes remain unclear.

Moreover, existing researches still have some limitations. Deep learning models are usually composed of multiple layers structures with neurons in each layer, which serve as the most vital hyperparameters in deep learning model and are particularly critical for the hierarchical FBNs. Hence, because of a variety of training parameters and the high dimensionality of fMRI volumes, it is difficult to manually design a suitable network architecture to model volumetric fMRI data, awaiting a computational framework to automatically construct the optimal neural architecture (NA). Furthermore, deep learning models that uncover the hierarchical FBNs and temporal patterns from high-dimensional NfMRI volumes usually lack ground truth and have to be trained in an unsupervised fashion, which are different from most existing NA search (NAS) methods designed for image classification problems (Pham et al., 2018; Real et al., 2019). To solve this problem, some studies have combined NAS framework with deep learning method and applied it to fMRI data to identify hierarchical FBNs (W. Zhang et al., 2019; Ren et al., 2021a,b). For instance, Ren and colleagues have proposed group-wise NAS-DBN to characterize the hierarchical spatiotemporal patterns from NfMRI volumes (Ren et al., 2021b). Specifically, these successful applications of DBN model on hierarchical FBN identification also reveal that a DBN model typically stacked by multiple restricted Boltzmann machine (RBM; Fischer and Igel, 2012) can naturally act as a hierarchical feature extractor to extract hierarchical brain spatiotemporal features.

Although the existing NAS-based frameworks can detect hierarchical spatiotemporal features (Dai et al., 2020; Li et al., 2021, 2022), there are still challenges. Previous studies reveal that though naturalistic paradigms trigger highly consistent brain responses across individuals, the neural temporal activities evoked by this condition also show great intersubject variability, especially in heteromodal association cortices, which reflects high degree of individuality and uniqueness in internal neural process (Golland et al., 2007; Ren et al., 2017b). However, previous models can only reveal the group-wise consistency among subjects, but cannot model the individual uniqueness (Qiang et al., 2020; Li et al., 2021). Moreover, it is not clear whether this property can be detected from volumetric NfMRI data (Y. Zhang et al., 2019). To address the challenges, we proposed a two-stage NAS-vsDBN model to automatically optimize NA and identify both group-consistent and individual-unique hierarchical spatiotemporal features from volumetric NfMRI data. Furthermore, we comprehensively validated the consistency and similarity between group-level and individual-level spatiotemporal features. Generally, based on the crucial property of NfMRI data, our model can effectively identify the hierarchical organization of brain function from NfMRI volumes.

## Materials and Methods

### Participants and video stimuli

Fifteen healthy subjects (eight females) participated in our study. Participants were interviewed to determine their eligibility through a telephone survey. We provided a complete and detailed description of our study and obtained written informed consent from all participants. The study was approved by the Yale Human Investigation Committee. In this experiment, we selected and presented a sad scenarios video as the input natural stimulation. In the video, an actress described the sad experience to the participants. More details of dataset and stimuli can be referred to previous studies (Kober et al., 2016).

### Imaging data acquisition and preprocessing

Participants were scanned in a 3 T Siemens Trio MRI scanner. The preprocessing process is implemented by the Data Preprocessing Assistant for Resting-state FMRI (DARSF) toolbox. The preprocessing pipeline includes head motion correction, slice time correction, spatial smoothing, band-filtering (0.008–0.3 Hz), and registration to MNI standard space. By calculating the spatial intersection of all individual brains, we generated a group-wise common mask to extract the whole-brain signals of each subject, by which we can ensure the spatial correspondence of each voxel across all the subjects. The extracted whole-brain fMRI signals of each subject were then stacked into a 2D matrix, where each column represents the fMRI signals of each voxel containing 206 time points, and each row refers to the brain volume possessing 70,831 voxels. In addition, fMRI time series of each voxel were normalized to have zero mean and unit variance. All individual subjects’ fMRI time series were concatenated, and then we transposed the connected matrix to a group-wise volumetric fMRI matrix for first stage DBN training.

### The overview framework of two-stage NAS-vsDBN model

**Figure 1.**
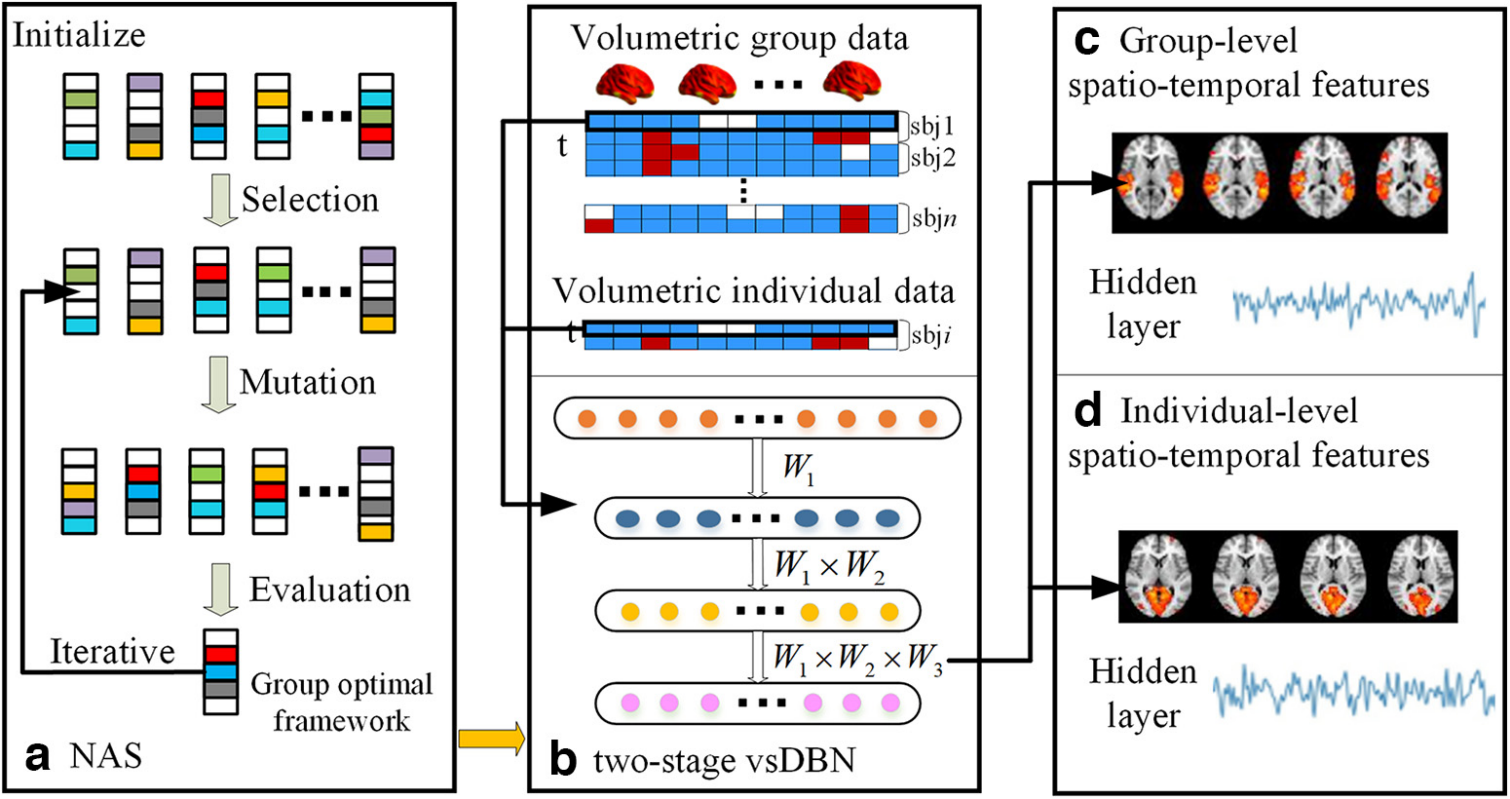
The computational framework of two-stage NAS-vsDBN model for whole-brain volumetric NfMRI signals, including (***a***) NAS framework, (***b***) Two-stage vsDBN model, (***c***) Group-level spatio-temporal features, and (***d***) Individual-level spatio-temporal features (NAS, neural architecture search; vsDBN, volumetric sparse deep belief network).

The two-stage NAS-vsDBN model we proposed is shown in Figure 1. We first performed 10 times NAS procedures on group-level NfMRI data. Specifically, we iteratively evaluated, mutated, and updated the randomly initialized subnets (Fig. 1*a*,*b*), resulting in the optimal NA for the group-wise vsDBN. Then the vsDBN model with the optimal NA was applied to train the group-level NfMRI data, after which the group-level spatiotemporal features were identified (Fig. 1*c*). Finally, we initialized the individual vsDBN model of second-stage training with the weights obtained from the first stage training to generate individual-level FBNs and temporal features with group consistency (Fig. 1*d*).

### NAS framework based on particle swarm optimization (PSO) algorithm

In order to search for the optimal network architecture (NA) of the two-stage vsDBN model, we adopted and designed a NAS framework based on the PSO algorithm. PSO is a representative and efficient swarm intelligent optimizer, which takes less time, has fewer parameters and is easy to implement (Kennedy and Eberhart, 1995). Specifically, we first randomly generated 30 subnets with different NAs, where each subnet was composed of the most vital hyperparameters for hierarchical organization characterization: the number of nodes and hidden layers. Subnets will be regarded as particles in PSO algorithm, which were then selected after initialization to perform mutations based on the idea of aging evolution to generate the new generations of subnets to increase the diversity of subnets (Real et al., 2019). The subnet after mutations will be mapped to the position of each particle. All the particles were evaluated by a fitness function, which was defined by the testing loss of vsDBN model (Kennedy and Eberhart, 1995). This NAS procedure was conducted iteratively. In the process of each iteration, the local best solution of each particle and the global best solution of whole swarm were recorded, and the original NA was replaced with the current global best NA with the minimum reconstruction error. Finally, after all the iterations, a single particle owning the most accurate reconstruction was selected as global optimal NA. Overall, all particles’ velocities and positions were updated by the following equations:

(1) $$v_{i}^{h+1}=w\text{ * }v_{i}^{h}+c_{1}\text{ * }Rand_{1}\left(pbest_{i}^{h}-nas_{i}^{h}\right)+c_{2}\text{ * }Rand_{2}(gbest_{i}^{h}-nas_{i}^{h})$$

(2) $$nas_{i}^{h+1}=nas_{i}^{h}+v_{i}^{h+1}.$$

Equations 1 and 2 are for mutative velocity and position updating, respectively, where $nas_{i}^{h}$ and $nas_{i}^{h+1}$ represent current and next search of NA, and $v_{i}^{h}$ and $v_{i}^{h+1}$ are the current and next velocities, respectively. The subscript *h* and *i* denote current iteration and subnet, respectively, w is the inertia weight that reflects the inertia of particle motion; $c_{1}$ and $c_{2}$ are constant real values; $Rand_{1}$ and $Rand_{2}$ are random real numbers selected from the interval [−1,1]; $pbest_{i}^{h}$ represents historical optima for each subnet during iterations, and $gbest_{i}^{h}$ is global optimal NA. The parameter settings of NAS are as follow: the constant values of w, $c_{1}$, and $c_{2}$ are, respectively, set as 0.1, 2, 2 according to previous study (Qiang et al., 2020; Ren et al., 2021b). Alternatively, as the value of w can be also updated by linearly decreasing weight method (Shi and Eberhart, 1998), we also repeated the NAS framework using this strategy. In the meanwhile, the search range of layers is set at [2, 10] and the search range of nodes is set to [100, 800].

### Two-stage vsDBN model of volumetric fMRI data

A DBN model consists of multiple stacked RBMs (Hinton et al., 2006). Inputs are modeled by RBMs via latent factors expressed through the interaction between hidden and visible variables (Hu et al., 2018). The stacked architecture of DBN model acts as a hierarchical feature extractor as a whole, generating an ideal model for extracting the hierarchical temporal and spatial features from high-dimensional volumetric NfMRI data. Specifically, a DBN model consists of visible layer variable v and hidden layer variable h, of which energy function is as follows:

(3) $$E\left(v,h\text{|}\theta \right)=-\overset{n}{\underset{i=1}{\sum }}a_{i}v_{i}-\overset{m}{\underset{j=1}{\sum }}b_{j}h_{j}-\overset{n}{\underset{i=1}{\sum }}\overset{m}{\underset{j=1}{\sum }}v_{i}W_{ij}h_{j}.$$

In the above formula, $\theta =\{W_{ij},a_{i},b_{j}\}$ represents the model parameters, where $W_{ij}$ is the connection weight between the visible layer unit i and the hidden layer unit j, $a_{i}$ represents the bias of the visible layer unit i, and $b_{j}$ represents the offset of the hidden layer unit j. After 10 times of NAS processes, we obtained the optimal network structure of DBN model, which was applied to define the architecture of group-wise vsDBN model. In this work, our optimal architecture of DBN has three RBM blocks. In the first stage, the individual NfMRI matrices of all the participants were first concatenated along time and then transposed, resulting in the group-wise volumetric NfMRI data matrix $F\in R^{\left(v\times n\right)\times m}$ ( v represents the number of fMRI time points, n is the number of individuals, and m represents the number of voxels in each fMRI volume, i.e., neurons in visible layer) for first-stage training. The optimized DBN was then applied to the group-level volumetric NfMRI data. After training, the weight matrices $W_{1}\in R^{m\times q_{1}}$, $W_{2}\in R^{\left(q_{1}\times q_{2}\right)}$, $W_{3}\in R^{\left(q_{2}\times q_{3}\right)}(q_{1},q_{2},q_{3}$ are the number of neurons in each hidden layer, respectively, and the value of $q_{1},q_{2},q_{3}$ are determined according to NAS process) between each hidden layer were learnt. In addition, we obtained the output of each hidden layer $T_{1}\in R^{\left(v\times n\right)\times q_{1}},T_{2}\in R^{\left(v\times n\right)\times q_{2}},T_{3}\in R^{\left(v\times n\right)\times q_{3}}$. In the second stage, we performed subject-specific vsDBN (ss-vsDBN) models using individual volumetric fMRI data to improve individual fMRI data representation, while retaining the spatiotemporal correspondence across subjects and groups. For this purpose, we used the optimal network structure and the weight matrices learned in the first stage ( $W_{1},W_{2},W_{3}$) to initialize ss-vsDBN. Specifically, $W_{1}$ was used to initialize the weights of the visible layer and the first hidden layer, and $W_{2},W_{3}$ were used to initialize the weights of the first and second hidden layer as well as the second and third hidden layer, respectively. Then, the initialized ss-vsDBN models were trained using each individual NfMRI data to obtain the individual-level weight matrices and temporal features ( $t_{1}\in R^{v\times q_{1}},t_{2}\in R^{v\times q_{2}},t_{3}\in R^{v\times q_{3}}$), respectively.

In previous FBNs identification studies, extracting spatiotemporal features from fMRI data using DBN/RBM model was regarded as a blind source separation problem (Hu et al., 2018; W. Zhang et al., 2019), which shared similar structures with matrix factorization problem in terms of the relationship among the observed fMRI data, latent temporal features, and spatial maps. Thus, volumetric NfMRI data can be decomposed as the temporal features and spatial maps through our vsDBN model. Specifically, for group-level and subject-level vsDBN model, the output of hidden layer represented temporal features, and the weight matrix of each layer represented the latent spatial features reflecting the extent of each voxel contributing to a latent variable, of which row can be directly mapped back into original 3D brain space to derive FBNs in a hierarchical manner. Specifically, the linear combination approach was used to extract the latent FBNs, where W 1 × W 2 × W three was visualized for the third hidden layer as FBNs and W 1 × W 2 and W 1 for the second hidden layer and the first hidden layer, respectively (Fig. 1*b*).

### Intersubject correlation (ISC) analysis for temporal features

Besides obtaining the hierarchical spatial features, we would like to further investigate the hierarchical organization of temporal responses derived from the two-stage NAS-vsDBN framework. Specifically, we calculated the ISC by using individuals’ temporal responses extracted from the output of each hidden layer, where ISC measured the intersubject consistency for temporal responses of each atom from each hidden layer across individuals (Hasson et al., 2004; Nastase et al., 2019). The ISC value was calculated for each atom of each hidden layer for all the subjects, separately. We then derived the ISC metric for each layer and each subject by averaging the ISC values across all the atoms belonging to the same layer, respectively. Moreover, the group-level ISC metric was derived by averaging all the individual’s ISCs for each layer, respectively.

### The spatial consistency between individual-level and group-level FBNs

The two-stage NAS-vsDBN model can identify the FBNs with both group consistency and individual uniqueness. Inspired by previous research, to further explore the spatial similarity between group-level and individual-level FBNs, we adopted the spatial correlation coefficient (SCC) as an indicator, which was defined in Equation 4 as $r_{gd}$

(4) $$r_{gd}=\frac{\sum_{\rho =1}^{p}(z_{g\rho }-\bar{z_{g}})(z_{d\rho }-\bar{z_{d}})}{\sqrt{\sum_{\rho =1}^{p}{\left(z_{g\rho }-\bar{z_{g}}\right)}^{2}\sum_{\rho =1}^{p}{\left(z_{d\rho }-\bar{z_{d}}\right)}^{2}}},$$

where $z_{g}$ represents the group-level FBNs identified from first stage vsDBN, and $z_{d}$ is the individual-level FBNs identified from second-stage vsDBN. $z_{g\rho }$ and $z_{d\rho }$ is *z score* value of p-th voxel of $z_{g}$ and $z_{d}$ $\bar{z_{g}}$ and $\bar{z_{d}}$ is the mean value of $z_{g}$ and $z_{d}$, respectively. p represents the total number of voxels in the whole brain, which refers to 70,831 in our experiment.

In addition to SCC, we also employed the overlap rate as a metric to explore the similarities/differences in spatial distribution between individual-level networks and corresponding group-level networks, where the spatial pattern overlap rate *R* was defined as:

(5) $$R\left(S,T\right)=\frac{\left|S\cap^{}T\right|}{\left|T\right|},$$

where S is the individual-level networks and T is corresponding group-level networks, respectively.

### The temporal consistency between individual-level and group-level dynamic functional connectivity (DFC)

Besides evaluating the spatial consistency of two-stage FBNs, we here further investigated the consistency of DFC derived from two-stage temporal responses. By the training of the two-stage NAS-vsDBN model, we have obtained both group-level ( $T_{3}^{i}$) and corresponding individual-level ( $t_{3}^{i}$) temporal features for each subject from the third layer of group-level and subject-level vsDBN model, respectively, where the group-level temporal features $T_{3}\in R^{\left(v\times n\right)\times q_{3}}$ can be also temporally divided into segments corresponding to each subject $T_{3}^{i}\in R^{v\times q_{3}}$ ( i is the index of the subjects), representing the individual-specific temporal features derived from group-level model. To explore whether the temporal features learned from two stages have consistent/different dynamic expressions, we derived all the subjects’ group-level and individual-level DFC, respectively. In detail, DFC was estimated with a commonly-used sliding window approach (Allen et al., 2014; Calhoun et al., 2014). Specifically, we first manually selected 43 meaningful networks from the third layer of group-level FBNs, and classified them into medial visual (V1), occipital pole visual (V2), lateral visual (V3), default mode (DM), sensorimotor (SM), auditory (Aud), executive control (EC), salience (SA) networks. Then, we selected both group-level and individual-level temporal features corresponding to these representative FBNs for sliding window analysis. Afterwards, for both group-level and individual-level temporal features, sliding time window approach was applied to obtain a series of windowed temporal features at different time points, and the windowed temporal features were then employed to measure the functional connectivity between each pair of representative FBNs within each corresponding time window $w_{i}(i=1...W$) using the Pearson correlation coefficient. Here, we used tapered window, created by the convolution of a rectangle (width = 22 TRs = 33 s) and Gaussian (σ = 3 TRs), with a step size of 2, resulting in W = 82 windows for each subject’s group-level and individual-level DFC. Based on the above steps, we can define both group-level and individual-level DFC matrix for each subject. Following this, a *k*-means clustering was both performed on the group-level and individual-level DFC patterns separately, to identify their representative connectivity “states,” where the *k* was set to four according to experience (Allen et al., 2014). Finally, to measure the similarity between group-level DFC states and individual-level DFC states, the similarity of DFC (SDFC) was defined by the following equation:

(6) $$SDFC=\overset{W}{\underset{i=1}{\sum }}\frac{1}{W}\times corr2\left(S_{ig},S_{id}\right),$$

where W represents the total number of windows, i is the index of the window, $S_{ig}$ and $S_{id}$ represent group-level and individual-level state matrices corresponding to the *i*-th window, respectively, and *corr2* calculates the Spearman rank correlation coefficient between two matrices used to measure the similarity between $S_{ig}$ and $S_{id}$.

### Code accessibility

The code of two-stage NAS-vsDBN framework described in the paper can be accessed in Extended Data 1.

10.1523/ENEURO.0200-22.2022.ed1 Extended Data 1 The code of two-stage NAS-vsDBN framework. Download Extended Data 1, ZIP file.

## Result

In this study, we proposed and applied a novel two-stage NAS-vsDBN model to model the volumetric NfMRI data, and examined the underlying hierarchical spatiotemporal organization of brain function. Overall, inspired by the inherent properties of neural process under natural viewing condition, our model aims to detect both group-consistent and individual-unique spatiotemporal features contained in NfMRI volume images. First, as designating suitable network architecture lays the foundation for extracting hierarchical organization of brain function, we performed 10 NAS processes independently to obtain the optimal NA of a reliable vsDBN model. Second, based on the optimized first-stage vsDBN model, we detected meaningful hierarchical group-level FBNs for the all subjects at multiple scales. In addition, by the second-stage vsDBN model training, the individual-level FBNs with both group consistency and individual uniqueness were obtained. Third, besides identifying both group-level and individual-level hierarchical spatial features, we further investigated the hierarchical organization of temporal responses using ISC analysis. Fourth, we calculated and compared the SCC between corresponding individual-level and group-level FBNs, so as to assess their consistency/difference in spatial features. Finally, we further investigated the consistency/difference of DFC derived from two-stage temporal responses.

### Two-stage NAS-vsDBN implementation

In order to quantitatively evaluate the effectiveness and stability of the NAS framework and obtain the optimal architecture for vsDBN model, we conducted 10 times NAS processes independently. We used group-wise NfMRI volume data as the training data set for NAS process.

**Figure 2.**
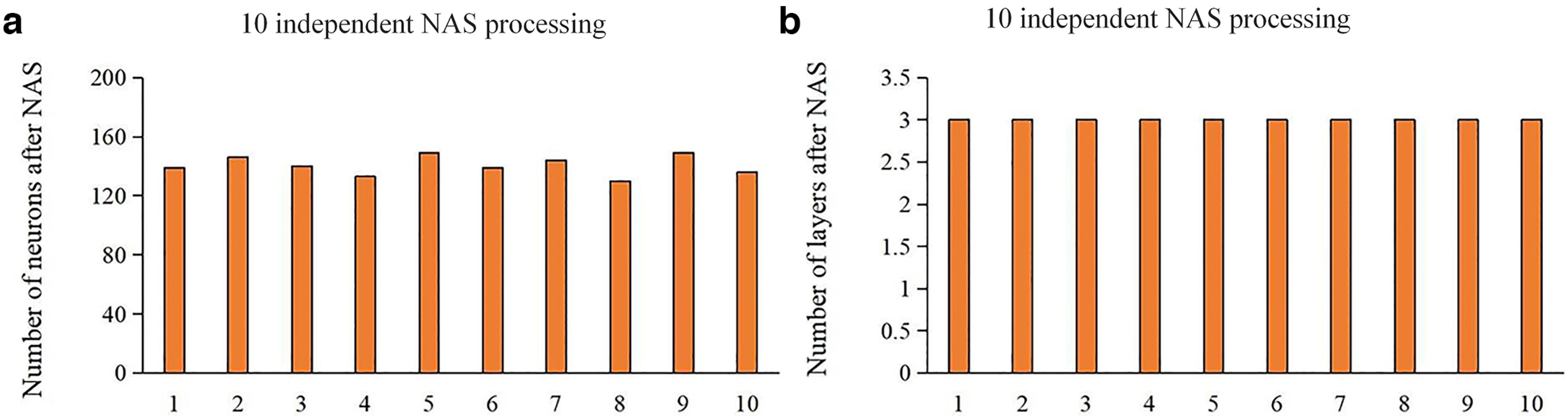
Results of 10 independent NAS processes. ***a***, Number of neurons after NAS. ***b***, Number of layers after NAS (NAS, neural architecture search). See also Extended Data Figures 2-1 and 2-2.

The NAS results showed that the number of neurons has been maintained between 120 and 150, and the number of layers were always three, which suggest our NAS process can generate highly consistent and robust NA (Fig. 2). In addition, we also repeated the NAS process using linear weight decreasing method, which yielded similar optimal NA and convergence rate (Extended Data Figs. 2-1, 2-2). After the optimization of NAS process, the reconstruction error of the third layer was <10^−5^. Thus, the optimal NA with the minimum reconstruction error among the 10 NAS results was selected, resulting in the optimal architecture of vsDBN model had three layers and 146 neurons. The optimized vsDBN model was further used to verify and obtain group-level and individual-level hierarchical spatiotemporal features of volumetric NfMRI.

10.1523/ENEURO.0200-22.2022.f2-1 Extended Data Figure 2-1 Results of five independent NAS processes using linearly decreasing weight. a, Number of neurons after NAS. b, Number of layers after NAS. Download Figure 2-1, TIF file.

10.1523/ENEURO.0200-22.2022.tab1-1 Extended Data Table 1-1 The p-values of two-sample t tests on the overlap rates among 8 representative FBNs (AUD, auditory; V1, medial visual; SM, sensorimotor; V2, occipital pole visual; DA, dorsal attention; SA, salience; DMN, default mode network; EC, executive control). Download Table 1-1, DOC file.

10.1523/ENEURO.0200-22.2022.f2-2 Extended Data Figure 2-2 Comparison of convergence speed between fixed weight and linearly decreasing weight. Download Figure 2-2, TIF file.

As vsDBN model takes a volume image from the NfMRI as a training sample and the volumetric fMRI image has 70 831 voxels, the number of visible units of the vsDBN in the first stage is 70 831. In addition, the number of neurons and layers of first-stage vsDBN is determined according to NAS process. Thus, the vsDBN model is composed of three layers of RBM, and the numbers of neurons in each layer ( $q_{1},q_{2},q_{3}$) are set to 146-146-146, respectively. The code was developed using the Deepnet framework (https://github.com/nitishsrivastava/deepnet) and ran on a deep learning server with GeForce GTX 1080 TI. The NAS process was accomplished on one GPU card within acceptable time (15 h).

### Hierarchical group-level and individual-level FBNs

**Figure 3.**
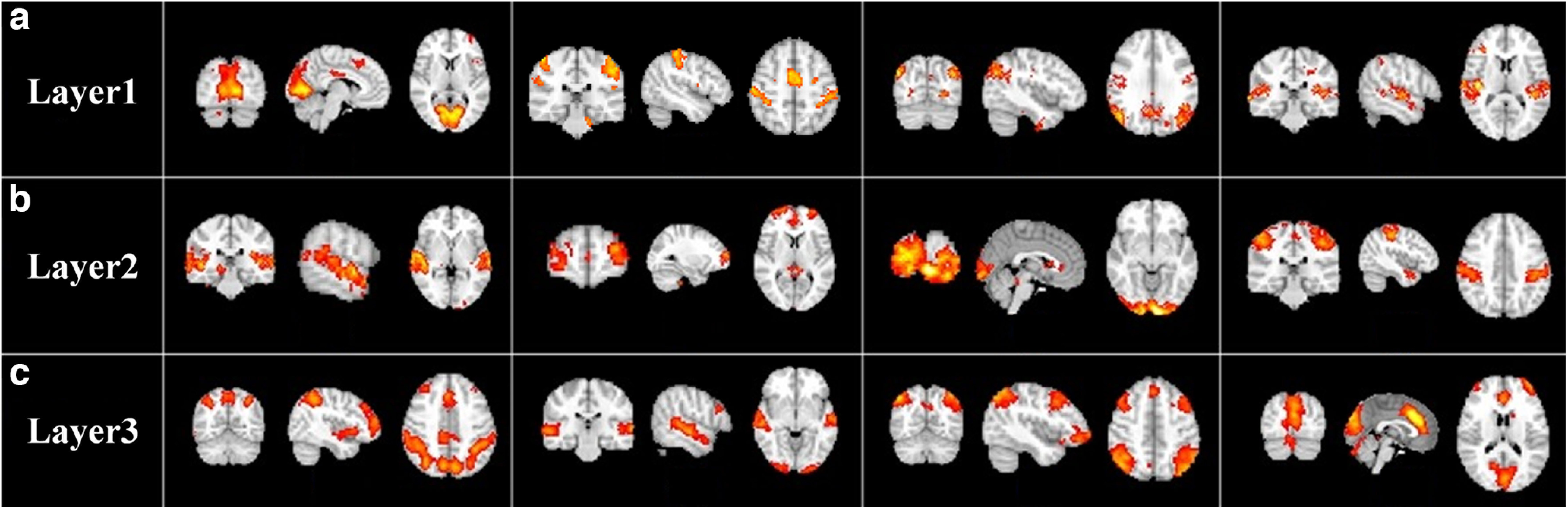
Representative group-level functional brain networks of (***a***) layer1, (***b***) layer2 and (***c***) layer3 identified by first-stage vsDBN model, respectively.

After identifying the optimal NA of vsDBN model by NAS framework, we first applied first-stage NAS-vsDBN model to obtain hierarchical group-level FBNs. Figure 3 shows the representative meaningful FBNs composed of several activated regions derived from each layer as example. In the first layer, there are medial-visual network, sensorimotor network, default mode network, auditory network (Fig. 3*a*). In the layer2, we detect auditory network, executive control network, occipital pole-visual network and sensorimotor network (Fig. 3*b*). Most of these networks identified in the shallow layer are related to primary sensory process, or simple and classic networks that are established well previously. However, some complex FBNs with several interacted brain regions/networks can be detected in deeper layer. In the layer3, there are executive control-dorsal attention network, occipital pole visual-auditory network, executive control-visual network (Fig. 3*c*). By comparing the FBNs identified from three layers of first-stage vsDBN model, while simple or primary FBNs are derived from shallower layers, networks obtained from deeper layer appear to be combinations of different functional networks/regions, which suggests the hierarchical organization of FBNs under naturalistic stimuli.

Previous literatures demonstrated that naturalistic stimuli can trigger highly consistent neural responses in primary sensory areas, however, neural activities excited by naturalistic condition in the higher-order heteromodal cortices reveal great intersubject variability (Golland et al., 2007; Ren et al., 2017a, 2021b). Thus, we used the second-stage vsDBN model to train individual volumetric

**Figure 4.**
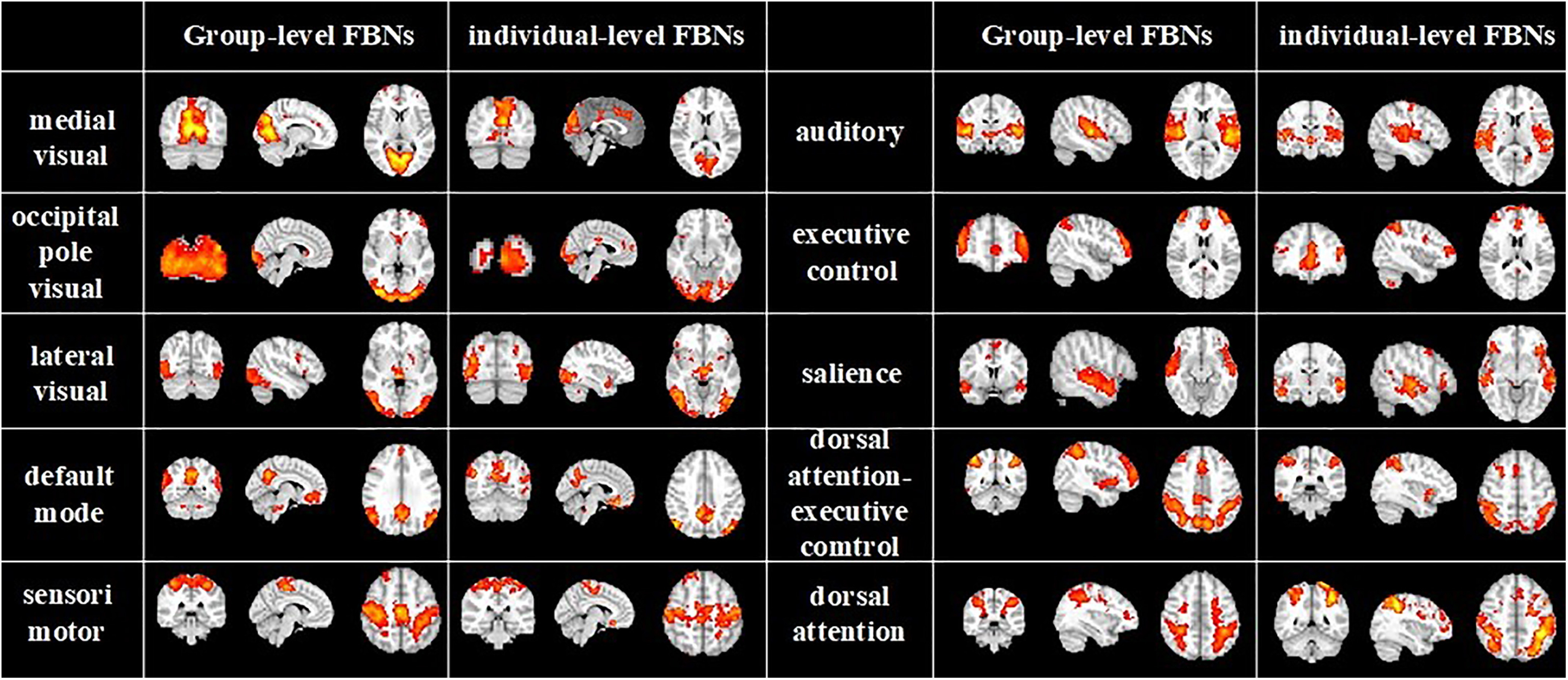
Representative group-level FBNs and corresponding individual-level FBNs in an exemplar subject (FBNs, functional brain networks).

fMRI data, and obtained individual-level FBNs. Specifically, to intuitively delineate the correspondence and difference between group-level and individual-level FBNs, we randomly selected a subject and compared its individual-level FBNs with group-level FBNs, including three visual networks, default mode network, sensorimotor network, auditory network, executive control network, salience network, dorsal attention-executive control network and dorsal attention network (Fig. 4). In general, the group-level and individual-level FBNs corresponds well, where the overall spatial patterns of the group identified from the first stage are well preserved in the individual-level results. However, the functional activations and region sizes of individual-level FBNs are different from group-level FBNs. These results reveal that our model can establish great correspondences between individuals and group in hierarchical spatial features, and can also maintain individual-specific variability.

### Hierarchical organization of temporal responses revealed by ISC analysis

**Figure 5.**
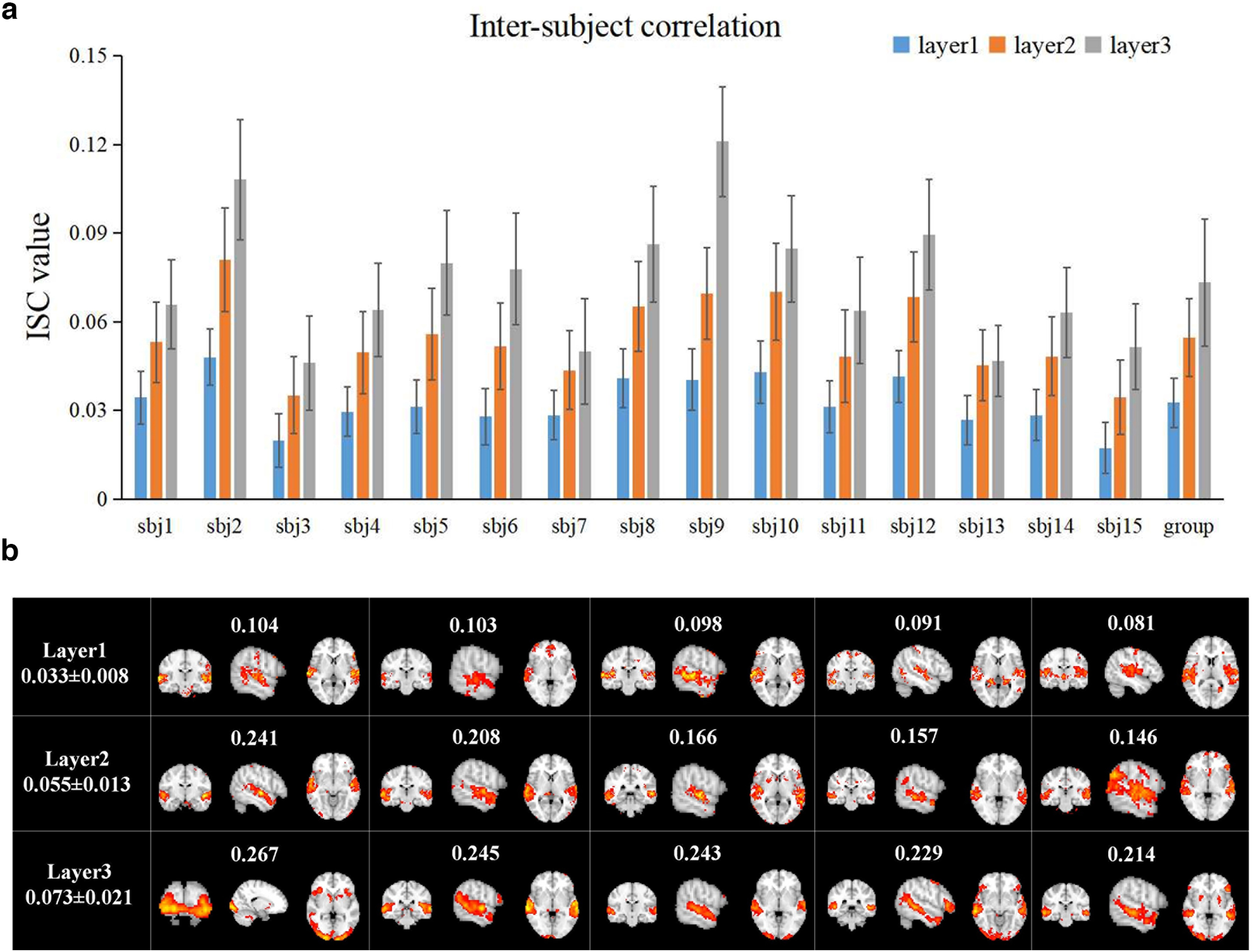
***a***, The ISC metrics in each layer at individual-level and group-level. Error bar indicates SD. ***b***, Top 5 group-level spatial maps with highest ISC metrics at each layer. ISC value corresponding to each FBN, and the average and SD ISC value of each layer are labelled (ISC: inter-subject correlation). See also Extended Data Figure 5-1.

In addition to exploring the hierarchical brain spatial features, we further investigated whether the proposed model can uncover the hierarchical structure of temporal responses from NfMRI volume images. Overall, we measured and compared the ISC of individual temporal responses for both individual-level and the group-level (Fig. 5*a*). The results indicate that compared with lower layer, the higher layer has higher ISC values at both individual and group level, which indicates that the higher-level temporal responses show higher intersubject consistency. Thus, the temporal features identified by the two-stage NAS-vsDBN model defer to a hierarchical structure under naturalistic paradigm.

10.1523/ENEURO.0200-22.2022.f5-1 Extended Data Figure 5-1 Comparison of group-level ISC values between two-stage tDBN model and two-stage NAS-vsDBN model. Error bar indicates SD. The statistical test was conducted by two-sample t test, where * represents FDR-corrected p < 1 × 1 × 10−3 and ** represents p < 1 × 1 × 10−4. Download Figure 5-1, TIF file.

Moreover, we also compared the ISC values of each FBN and selected the top five FBNs with the highest group-level ISC in each layer (Fig. 5*b*). Generally, these top FBNs are mostly composed of visual, auditory and primary sensory regions/networks, suggesting that temporal features related to primary sensory processes have higher intersubject consistency driven by external naturalistic stimuli. However, FBNs associated with higher-order association regions at the third layer, such as prefrontal cortex and anterior cingulate cortex, also reveal high consistency across subjects. These association regions are not commonly associated with sensory processing, which further demonstrates the capability of proposed model in extracting hierarchical temporal features.

### The consistency in spatial features between individual-level FBNs and group-level FBNs

**Figure 6.**
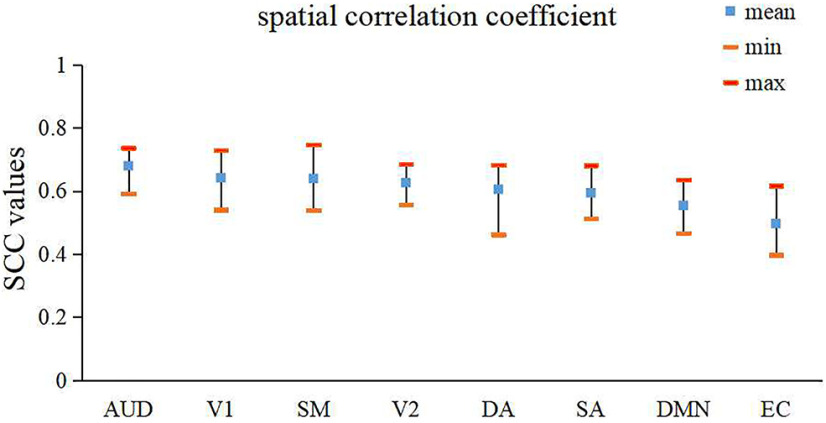
The spatial correlation coefficient (mean, minimum and maximum) between each individual-level FBN and corresponding group-level FBN (SCC, spatial correlation coefficient; AUD, auditory; V1, medial visual; SM, sensorimotor; V2, occipital pole visual; DA, dorsal attention; SA, salience; DMN, default mode network; EC, executive control). See also Extended Data Figures 6-1, 6-2, and 6-3.

The experimental results in the previous sections show that our two-stage NAS-vsDBN framework can well identify hierarchical spatiotemporal features and establish the correspondence of spatial distribution between individuals and groups. Nonetheless, the individual-level FBNs also retain their uniqueness in functional activation. Therefore, we here further quantitatively explored and assessed the similarity/differences in spatial features between individual-level networks and the corresponding group networks. Specifically, we selected 8 most representative group-level FBNs identified in the first-stage vsDBN for comparison, including auditory network, medial-visual network, occipital pole-visual network, sensorimotor network, dorsal attention network, executive control network, default mode network and salience network. We thus calculated the SCC values between individual-level and corresponding group-level FBNs as a measurement of similarity in spatial features (Fig. 6). It can be seen those FBNs that are associated with primary sensory processes, including auditory network, medial-visual network, sensorimotor network and occipital pole-visual network, exhibited higher overlap rates (0.68, 0.64, 0.63, 0.62, respectively). In comparison, for those networks associated with higher-order cognitive processes or emotional perception, such as salience network, default mode network and executive control network, there are significant decreases in their overlap rates (0.59, 0.55, 0.49, respectively).

10.1523/ENEURO.0200-22.2022.f6-1 Extended Data Figure 6-1 The overlap rate (mean, minimum, and maximum) between each individual-level FBN and corresponding group-level FBN (V1, medial visual; AUD, auditory; V2, occipital pole visual; SM, sensorimotor; DA, dorsal attention; SA, salience; DMN, default mode network; EC, executive control). Download Figure 6-1, TIF file.

10.1523/ENEURO.0200-22.2022.f6-2 Extended Data Figure 6-2 Comparison of the SCC between two-stage tDBN model and two-stage NAS-vsDBN model. Error bar indicates SD. The statistical test was conducted by two-sample t test, where * represents p < 1 × 1 × 10−2 (AUD, auditory; V1, medial visual; SM, sensorimotor; V2, occipital pole visual; DMN, default mode network; EC, executive control). Download Figure 6-2, TIF file.

To further quantitively evaluate the differences in SCC among different networks, we performed two-sample *t* tests with false discovery rate (FDR)-corrected (*p* < 0.01) on the overlap rate values among eight FBNs, and the *p*-value were shown in Table 1. The statistical results show that the SCC values of salience network, default mode network and executive control network are significantly lower than those of auditory networks (*p* < 1 × 10^−4^). In addition, the SCC of executive control network is significantly lower than those of visual network, sensorimotor network, dorsal attention network and salience network (*p* < 1 × 10^−4^). These results indicate that the networks related to primary sensory regions have higher consistencies between individual-level and group-level FBNs, while networks involved in cognitive or emotional perception processes show stronger intersubject variabilities in spatial distributions, further supporting the underlying hypothesis of our two-stage vsDBN model. In addition, we also calculated the overlap rates between individual-level and corresponding group-level FBNs, which produces similar results as SCC analyses and basically reflect the same conclusion. Therefore, we presented the results based on overlap rate in the Extended Data as a further validation (Extended Data Fig. 6-1; Extended Data Table 1-1).

**Table 1:** *P*-values of two-sample *t* tests on the SCC among eight representative FBNs

	AUD	V1	SM	V2	DA	SA	DM	EC
AUD								
V1	0.056							
SM	0.034	1						
V2	9 × 10^−4^	0.464	0.504					
DA	1 × 10^−3^	0.147	0.153	0.356				
SA	1 × 10^−4^	0.038	0.038	0.088	0.708			
DM	6 × 10^−8^	1 × 10^−4^	1 × 10^−4^	1 × 10^−4^	0.034	0.056		
EC	8 × 10^−10^	4 × 10^−7^	3 × 10^−7^	2 × 10^−7^	1 × 10^−4^	1 × 10^−4^	0.009	

AUD, auditory; V1, medial visual; SM, sensorimotor; V2, occipital pole visual; DA, dorsal attention; SA, salience; DMN, default mode network; EC, executive control. See also Extended Data Table 1-1.

### The temporal consistency between individual-level and group-level DFC

**Figure 7.**
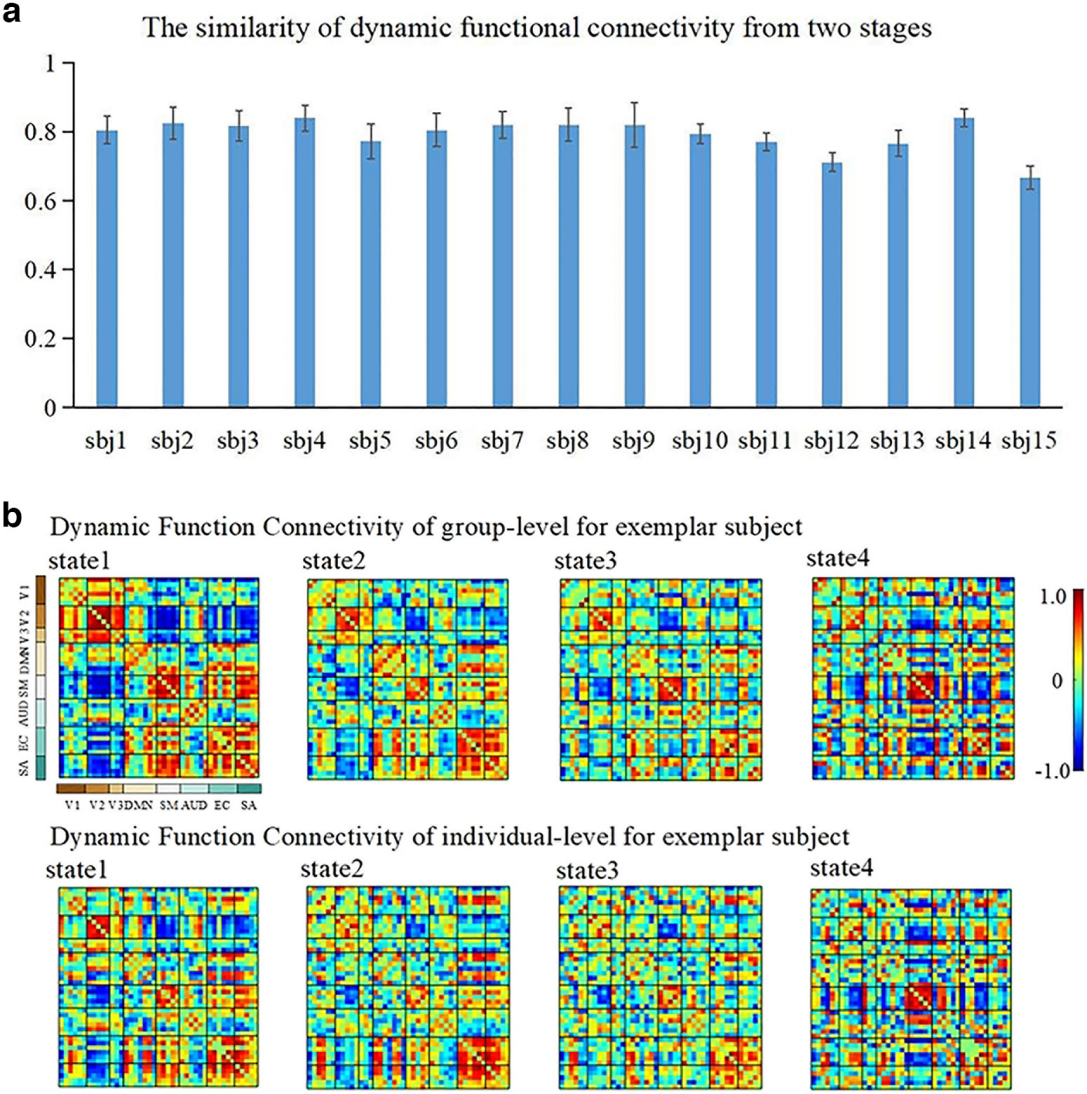
***a***, The dynamic functional connection similarity (SDFC) metrics between individual and group levels. Error bar indicates SD. ***b***, Representative DFC states at group-level and individual-level for an exemplar subject (sbj, subject; V1, medial visual; V2, occipital pole visual; V3, lateral visual; DMN, default mode network; SM, sensorimotor; AUD, auditory; EC, executive control; SA, salience).

Based on the existing experimental results, we find that the group-level FBNs and individual-level FBNs identified by proposed model maintain great consistency in spatial distributions, while individual-level FBNs retain their uniqueness in functional activations and spatial distributions. Besides this, we want to further explore whether the temporal features extracted from two stages can maintain consistency. To measure the similarity of temporal features identified from the two stages, we calculated the DFC at the group-level and individual-level, and then obtained four representative functional connectivity states at both group-level and individual-level for each individual, respectively. Then we calculated and summarized the SDFC of each individual as shown in Figure 7. According to Figure 7*a*, the SDFC values of all the individuals exceed 0.6, and more than half of individuals’ SDFC exceeded 0.8, which indicating that temporal features extracted from the two stages maintain great consistency. These results also further indicate the effectiveness of proposed model in identifying reliable temporal features from volumetric NfMRI data.

We further illustrated the correspondence between representative DFC states of the group-level and individual-level by randomly selecting a subject as an example (Fig. 7*b*). According to the order of occurrence of each DFC state, we marked the clustering states as states 1–4. The DFC states derived from group-level and individual-level show high consistency in functional connectivity network topology, where the correlations within visual networks and sensorimotor networks are mostly positive and strong among all the states, and the correlations within and between executive control and salience networks has also have strong and positive values. In addition, we calculate the correlation coefficients between the corresponding state matrices illustrated in Figure 7*b*, and the values are 0.79, 0.88, 0.73, and 0.82, respectively, which suggests high consistency between DFC states at group-level and individual-level.

## Discussion

In this paper, we proposed and applied a novel two-stage NAS-vsDBN model to uncover the hierarchical organization of spatiotemporal features from volumetric NfMRI data. Our main work is summarized as follows. First, we proposed NAS framework based on PSO, which can automatically construct a feasible optimal network structure for the vsDBN model under limited computing resources and an acceptable time. Based on the established optimal NA, the proposed two-stage vsDBN model can effectively reveal the hierarchical organization of spatial patterns and temporal responses with both group-consistency and individual-uniqueness properties. Specifically, different from the simple NAS-DBN model, our two-stage NAS-vsDBN framework has been established and developed based on the critical properties of naturalistic paradigm, that is, while this paradigm trigger highly consistent brain responses in primary sensory areas across subjects, the neural activities evoked by this paradigm also show great intersubject variability, especially in heteromodal association cortices (Golland et al., 2007; Ren et al., 2017b). Consequently, while the simple NAS-DBN model can only identify group-level spatial patterns and the individual-level variations across subjects might be overlooked, our framework could establish correspondence between two stages of vsDBN models. Second, as previous literature revealed that volatile fMRI time series possess more intersubject variability compared with spatial volumes in different imaging sessions (Schmithorst and Holland, 2004), by systematic comparisons between our proposed two-stage vsDBN and a two-stage temporal DBN (two-stage tDBN) with same training parameters and network architecture, we further verified the potential superiority of volumetric fMRI in identifying brain functional spatiotemporal features. Specifically, the comparative results demonstrated that ISC values of identified temporal features, and SCC and spatial overlap values between group-level FBNs and individual-level FBNs of vsDBN are superior than those metrics derived from two-stage tDBN (Extended Data Figs. 5-1, 6-1, 6-2), further supporting the adoption of volumetric fMRI data for modeling functional spatiotemporal features. Third, based on further comparison of the consistency/difference in spatial distribution between group-level and individual-level FBNs, our experimental results demonstrate that functional networks/regions related to cognitive or emotional perception processes exhibited greater intersubject variability than primary sensory associated regions such as visual and auditory networks, further verifying the hypothesis of proposed model. Finally, by calculating individual-level and group-level DFC from temporal features and measuring the SDFC between them, we found that the temporal features identified from the two-stage model show high similarity. Overall, our experimental results indicate the superiority and effectiveness of our model in uncovering hierarchical spatiotemporal features from NfMRI volumes that conform to the most critical property of neural process during natural viewing condition.

In the future work, we will focus on improving the efficiency of our NAS optimization framework and applying our model to datasets with larger sample size and richer types of naturalistic stimuli, and further investigating the hierarchical spatiotemporal organization of brain function under natural viewing condition. Furthermore, we a expect that in the near future our framework can be applied to clinical populations to characterize the abnormal brain function and develop neuroimaging markers under naturalistic condition.

10.1523/ENEURO.0200-22.2022.f6-3 Extended Data Figure 6-3 Comparison of the overlap rate between two-stage tDBN model and two-stage NAS-vsDBN model. Error bar indicates SD. The statistical test was conducted by two-sample *t* test, where * represents *p *<* *1 × 1 × 10^−2^ (V1, medial visual; V2, occipital pole visual; AUD, auditory; SM, sensorimotor; DMN, default mode network; EC, executive control). Download Figure 6-3, TIF file.

## Synthesis

Reviewing Editor: Niraj Desai, National Institute of Neurological Disorders and Stroke Decisions are customarily a result of the Reviewing Editor and the peer reviewers coming together and discussing their recommendations until a consensus is reached. When revisions are invited, a fact-based synthesis statement explaining their decision and outlining what is needed to prepare a revision will be listed below. The following reviewer(s) agreed to reveal their identity: Xi Jiang, Fateme Ghayem.

Two scientists have reviewed the manuscript. We all agree that the work has merit, but the reviewers raised some important concerns (see the reviews below). Please respond point-by-point and in detail when you resubmit.

REVIEWER # 1

Major concerns:

1. The authors propose a two-stage NAS-vsDBN model and provide deepnet code. The function of each provided file is not explained nor the function of each provided file. The code is not well commented and has paths hardcoded that would need to be modified.

2. One important contribution of this paper is to extract the individual-level FBNs with group-wise correspondence from volumetric fMRI data. What are the advantages of using volumetric fMRI data compared with fMRI time series? Please add additional experiments and clarify it.

3. The literature of identifying FBNs using deep learning model is probably being downplayed. The authors could include more relevant references especially those based on NAS in the introduction part.

4. Is the proposed two-stage NAS-vsDBN better than the vanilla NAS-DBN? If yes, could the author provide more information to illustrate that the two-stage NAS-vsDBN is better than that vanilla NAS-DBN?

Minor:

5. How is the computation efficiency of the proposed model?

6. In Figure 1, the label of each subfigure should be lowercase letters.

7. There are many kinds of deep models that could be used to characterize the FBNs as the authors mentioned in introduction, why do author choose DBN?

8. The full name of the abbreviation presented in the figure should be provided accurately. EX, ‘DMN’ in Fig. 7b.

9. Please double check the format of references.

REVIEWER #2

1- On the proposed model:

* The difference between this work and the following published paper is not clear and they are highly overlapped:

A two-stage DBN-based method to exploring functional brain networks in naturalistic paradigm fMRI. Paper presented at the 16th IEEE International Symposium on Biomedical Imaging (ISBI).

* For the PSO algorithm it is suggested to start with a large inertia weight w, and gradually decrease it, while the authors are using a fixed value w=0.1. This decreases the convergence rate.

*How many iterations were required for the training phase? It can be useful to mention the time required to achieve the final results.

* Required to report q1, q2, and q3.

2- On dataset:

* Are all 15 subjects of healthy control?

* Yet 15 subjects is very small.

* What is the number of voxels?

3- On metrics:

* Spatial pattern overlap rate R defined in eq. (4) does not seem to well measure the similarities between individual level and group level networks by taking the intersection in the numerator. Isn’t Pearson correlation a better measure? How about other metrics in this application?

4- Typo:

Line 210 and 211, is it W1*W2*W3 or W3*W2*W1 to be visualized?

The same about W1*W2.

## Author Response

We greatly appreciate the reviewers’ constructive comments and suggestions that have guided us to improve the quality of the paper tremendously. Our responses are itemized and detailed as follows. Attached is the revised version of our manuscript with revisions highlighted.

REVIEWER #1

Major concerns:

1. The authors propose a two-stage NAS-vsDBN model and provide deepnet code. The function of each provided file is not explained nor the function of each provided file. The code is not well commented and has paths hardcoded that would need to be modified.

Author response (AR): We apologize for the confusion of the code in the previous submission. We have now revised each code file into function and removed the paths hardcoded in the code. To further improve the readability of code, we have added comments for each the code in the revised version.

2. One important contribution of this paper is to extract the individual-level FBNs with group-wise correspondence from volumetric fMRI data. What are the advantages of using volumetric fMRI data compared with fMRI time series? Please add additional experiments and clarify it.

AR: Thanks for the suggestions. While recent studies have already explored the extraction of spatio-temporal features from fMRI time series (W. Zhang et al., 2019; Y. Zhang et al., 2019), previous literature revealed that inter-subject variability across different subjects is relatively more associated with volatile fMRI time series compared with spatial volumes in different imaging sessions (Schmithorst & Holland, 2004). Thus, it appears that taking volumes as input possibly works better than time series in terms of modeling the hierarchical functional brain networks (FBNs) for fMRI data in this case (Dong et al., 2020; Qiang et al., 2020; Schmithorst & Holland, 2004).

In order to further examine the potential advantages of using volumetric fMRI for modeling spatio-temporal features, we applied a two-stage temporal deep belief network (two-stage tDBN) on the same dataset to characterize spatio-temporal features from fMRI time series, in which the network architecture (NA) and training parameters were consistent with our proposed two-stage NAS-vsDBN (neural architecture search and volumetric sparse deep belief network) model. Specifically, we first calculated and compared the group-level inter-subject correlation (ISC) values of the temporal features identified by the two different models (Figure 1). According to Figure 1, we can observe that the ISC values of each layer of vsDBN model are significantly higher than tDBN (two-sample t-test, False discovery rate corrected p < 1×10-3), indicating better inter-subject consistency for temporal responses derived from volumetric-based model. In addition, we then calculated and compared the spatial correlation coefficient (SCC) and the spatial overlap rate between group-level FBNs and individual-level FBNs obtained from two comparative models, respectively, which assessed the performance of two models in extracting meaningful and consistent FBNs (Figure 2 and Figure 3). The experimental results demonstrate that both SCC and overlap rate of vsDBN model are higher than tDBN model in all the representative networks, where significant differences in sensorimotor network and executive control network can be found for both two metrics (p < 1×10-2). These experimental results show the advantages of modeling volumetric fMRI data in identifying meaningful FBNs and associated temporal features. According to this suggestion, we have supplemented the relevant experimental results in the Extended Data (Figure 5-1, 6-1 and 6-2) and added additional discussions in the Discussion part (Line 475 to 485 “Second, as previous literature revealed ...”).

Figure 1. Comparison of group-level ISC values between two-stage tDBN model and two-stage NAS-vsDBN model. Error bar indicates standard deviation (S.D.). The statistical test was conducted by two-sample t-test , where * represents FDR-corrected p < 1×10-3 and ** represents p < 1×10-4 (FDR: False discovery rate) .

Figure 2. Comparison of the spatial correlation coefficient (SCC) between two-stage tDBN model and two-stage NAS-vsDBN model. Error bar indicates standard deviation (S.D.). The statistical test was conducted by two-sample t-test, where * represents FDR-corrected p < 1×10-2 (AUD, auditory network; V1, medial visual; SM, sensorimotor; V2, occipital pole visual; DMN, default mode network; EC, executive control).

Figure 3. Comparison of the overlap rate between two-stage tDBN model and two-stage NAS-vsDBN model. Error bar indicates standard deviation (S.D.). The statistical test was conducted by two-sample t-test, where * represents FDR-corrected p < 1×10-3 (V1, medial visual; V2, occipital pole visual; AUD, auditory; SM, sensorimotor; DMN, default mode network; EC, executive control).

3. The literature of identifying FBNs using deep learning model is probably being downplayed. The authors could include more relevant references especially those based on NAS in the introduction part.

AR: Thanks for the suggestions. We agree that we should include comprehensive relevant literature in the introduction part. To make the existing relevant studies clearer, we have added additional relevant literature listed below in the Introduction part (Line 87).

Li, Q., Wu, X., & Liu, T. M. (2021). Differentiable neural architecture search for optimal spatial/temporal brain function network decomposition. Medical Image Analysis, 69, 14.

Dai, H. X., Ge, F. F., Li, Q., Zhang, W., Liu, T. M., & Ieee. (2020). Optimize CNN model for fMRI signal classification via adanet based neural architecture search. Paper presented at the IEEE 17th International Symposium on Biomedical Imaging (ISBI).

4. Is the proposed two-stage NAS-vsDBN better than the vanilla NAS-DBN? If yes, could the author provide more information to illustrate that the two-stage NAS-vsDBN is better than that vanilla NAS-DBN?

AR: Thanks for these comments. Compared to vanilla NAS-DBN model, the advantages of our two-stage NAS-vsDBN can be summarized as follows. Specifically, different from the simple NAS-DBN model, our two-stage NAS-vsDBN framework has been established and developed based on the critical properties of naturalistic paradigm, that is, while this paradigm trigger highly consistent brain responses in primary sensory areas across subjects, the neural activities evoked by this paradigm also show great inter-subject variability, especially in heteromodal association cortices (Golland et al., 2007; Y. Ren et al., 2017). Consequently, while the simple NAS-DBN model can only identify group-level spatial patterns and the individual-level variations across subjects might be overlooked, our framework could establish correspondence between two stages of vsDBN models. Specifically, vanilla NAS-DBN can be divided into two categories: (1) Employing volumetric fMRI data as sample, the model is the same as the first-stage DBN of our model. directly extracting hierarchical structures of group-level FBNs from fMRI volume (Dong et al., 2020; Qiang et al., 2020). (2) Adopting fMRI time series as sample of DBN model, the model can yield hierarchical temporal features and associated group-level FBNs (W. Zhang et al., 2019). As these two kinds of vanilla NAS-DBN model can only identify group-level spatial patterns, the individual-level variation might be overlooked and the motivation is different from that of our model.

Furthermore, based on the well-established correspondence between two stages of vsDBN models, our framework could offer an effective tool for inter-group/subject comparison of representative temporal features/FBNs, especially for clinical populations. Thus, our two-stage NAS-vsDBN framework holds important implications for clinical applications, to elucidate abnormal brain function and develop neuroimaging markers for neuropsychiatric disorders. To help readers understand the advantages of our model better, we have included additional descriptions in the Discussion part (Line 466-475 “Specifically, different from the simple NAS-DBN model ...”) of the new manuscript to clarify.

Minor:

5. How is the computation efficiency of the proposed model?

AR: Thanks for the suggestions. Previous research literature revealed that NAS process for fMRI analyses usually need plenty of computational resources (Li et al., 2021; Qiang et al., 2020). In our study, despite the high dimensionality of NfMRI data and unsupervised training of model, our NAS process was implemented on one GPU card (GeForce GTX 1080 TI) within an acceptable time (15 hours), which was comparable with the computation efficiency of NAS frameworks for fMRI analyses in previous literature (Li et al., 2021; Qiang et al., 2020). We have provided detailed information in Line 335 to 336 “The NAS process was ...”.

6. In Figure 1, the label of each subfigure should be lowercase letters.

AR: Thanks for pointing out this. We have corrected the figure and updated it in the current version.

7. There are many kinds of deep models that could be used to characterize the FBNs as the authors mentioned in introduction, why do author choose DBN?

AR: Thanks for the suggestion. We agree with the reviewer that there exist various kinds of deep learning models that could be applied to identify FBNs. However, we here chose DBN model to develop our framework and characterize hierarchical FBNs for reason. Previous research studies demonstrated that hierarchical processing is a key principle of neural computation in the brain (Park and Friston, 2013), thus researchers have long been looking forward to employing suitable deep learning model to uncover the intrinsic hierarchical organization of brain activities and networks. Recently, there have been several studies based on DBN model that can effectively characterize the hierarchical functional activations from fMRI data (W. Zhang et al., 2019; Dong et al., 2020; Qiang et al., 2020; Y Ren et al., 2021a; Y Ren et al., 2021b). For instance, Zhang et al has developed a NAS framework (Hybrid Spatiotemporal NASNet, HS-NASNet) based on particle swarm optimization (PSO) to model tb-fMRI signals, whose main idea is that the particle swarm in the NAS framework will evolve along time and eventually converge to a feasible optimal solution (W. Zhang et al., 2019). Moreover, Ren et al has proposed group-wise NAS-DBN to characterize the hierarchical spatiotemporal patterns from NfMRI volume (Y Ren et al., 2021b) Specifically, these successful applications of DBN model on hierarchical FBN identification also reveal that a DBN model typically stacked by multiple Boltzmann machine (RBM) (Fischer and Igel, 2012) can naturally act as an effective hierarchical feature extractor as a whole to extract hierarchical brain spatio-temporal features. To help readers understand our idea better, we have added Line 77 to 85 “To solve this problem, some studies have successfully ...” in the new manuscript to provide more explanations and make our point.

8. The full name of the abbreviation presented in the figure should be provided accurately. EX, ‘DMN’ in Fig. 7b.

AR: Thanks for the reminder. We have now double-checked the manuscript and provided correct full names for the all the abbreviations.

9. Please double check the format of references.

AR: We apologize for the format error of references in previous submission. We have now double-checked and reformatted all the references.

REVIEWER #2

1- On the proposed model:

* The difference between this work and the following published paper is not clear and they are highly overlapped:

A two-stage DBN-based method to exploring functional brain networks in naturalistic paradigm fMRI. Paper presented at the 16th IEEE International Symposium on Biomedical Imaging (ISBI).

AR: We apologize for the confusion. The literature published on 16th IEEE International Symposium on Biomedical Imaging (ISBI) mainly proposed a two-stage temporal DBN framework with manually defined network architecture to identify functional brain networks by modeling NfMRI time series. There are three main differences between our research and this literature. First, the neural architecture (NA) of DBN model was manually defined by experience in the mentioned literature. Due to the high dimensionality of fMRI data and a variety of training parameters, manual design of neural architecture depending on experience is very time-consuming and less reliable, further affecting the characterization of hierarchical FBNs. Therefore, we here proposed a neural architecture search (NAS) framework, which can find a feasible optimal solution of neural architecture for vsDBN model within acceptable time with limited computing resource. Second, as previous literature revealed that inter-subject variability across different subjects is relatively more associated with volatile fMRI time series compared with spatial volumes in different imaging sessions, taking volumes as input might works better than time series in terms of modeling the FBNs for fMRI data. (Dong et al., 2020; Qiang et al., 2020; Schmithorst & Holland, 2004) Thus, the main purpose of our study is to identify hierarchical spatio-temporal features of brain function by modeling volumetric fMRI data, where vsDBN model with the optimal NA was applied. However, the previous study employed a two-layer DBN model, which was applied to model NfMRI time series and cannot be used to uncover hierarchical spatio-temporal features. Finally, while the mentioned literature and our study proposed two-stage DBN frameworks using NfMRI time series and volumes respectively, our study provided more systematic and comprehensive evaluations of difference/consistency between group-level and individual-level spatio-temporal features, including spatial correlation coefficient (SCC), spatial overlap rate, inter-subject correlation (ISC) and similarity of dynamic functional connectivity (SDFC), of which results further verified the underlying hypothesis of our two-stage vsDBN model.

In summary, we believe that there are essential differences and significant improvements between our study and the literature mentioned. In order to help readers understand our idea better, we have added details descriptions in Line 94 to 95 “Moreover, it has been rarely investigated...”

* For the PSO algorithm it is suggested to start with a large inertia weight w, and gradually decrease it, while the authors are using a fixed value w=0.1. This decreases the convergence rate.

AR: We greatly appreciate the suggestions. According to the suggestion, we further examined whether the initialization and selection of inertia weight w will affect the convergence speed and results of our NAS process. Specifically, we used a linearly decreasing weight method (Shi & Eberhart, 1998) to continuously reduce the value of w (initial w = 0.5) in the iteration of NAS process, and independently repeated the NAS process on the same dataset for five times. Consequently, the NAS results showed that the number of neurons was maintained between 130 and 150 (140.6{plus minus}8.14), and the number of layers was always three, which was quite consistent with the results using fixed weight (Figure. 5). In addition, it took almost the same time to apply these two different weight strategies to execute a NAS process, because the training process of vsDBN consumed much more time in the NAS process. These experimental results show that the fixed weight method can accurately build the optimal neural architecture (NA) for our vsDBN model.

Figure 5. Results of 5 independent NAS processes using linearly decreasing weight. (a) Number of neurons after NAS. (b) Number of layers after NAS.

Moreover, we compared the convergence speed of the two weight selection strategies. According to Figure 6, we find that the converge speed to the final optimal result of linearly decreasing weight method is quite similar to the fixed weight method (both weight strategies converge at the 3rd to 5th iteration), further supporting the selection of a fixed inertia weight w in our NAS process.

However, as suggested by the reviewer and previous literature (Ferrarini et al., 2009; Shi & Eberhart, 1998, 1999), a larger inertia weight can enhance the global search ability to avoid falling into the local optimal solution and reduce the convergence speed. Therefore, in future, we will improve our algorithm to increase the efficiency of our NAS framework. To help readers understand our idea better, we have added these experiment results in Extended Data (Fig. 2-1, 2-2) and detailed descriptions in Line 168 to 170 “Alternatively, as the value of w can ...”, in Line 320 to 322 “In addition, we also repeated the NAS” and in Line 496-499 “In the future work, we will focus on Improving the efficiency ...”in the updated manuscript.

Figure 6. Comparison of convergence speed between fixed weight and linearly decreasing weight strategies.

How many iterations were required for the training phase? It can be useful to mention the time required to achieve the final results.

AR: We greatly appreciate the suggestions. In general, the proposed framework includes two main parts: NAS process (repeated 10 times) and the training of two-stage vsDBN model. Specifically, in one NAS process, we initialized 30 sub-nets and performed mutations on selected sub-nets, then performed vsDBN training for each sub-net in one iteration, and finally assessed whether to update the sub-net according to its reconstruction error after training. In one NAS process, we need to perform 10 iterations. Overall, a complete NAS process takes 15 hours with GeForce GTX 1080 TI. We have provided detailed information about the time consumption in Line 335 to 336 “The NAS process was ...”.

* Required to report q1, q2, and q3.

AR: Thanks for the suggestion. q_1,q_2,q_3 are the number of neurons in each hidden layer, respectively. According to the optimal NA derived by 10 NAS processes, we finally set the values of q_1,q_2,q_3 to 146-146-146. To help readers understand our idea better, we have added detailed descriptions in Line 195 to 196 (“and the value of q_1,q_2,q_3 are”) and Line 332 to 333 (“and the numbers of neurons...”).

2- On dataset:

* Are all 15 subjects of healthy control?

AR: Sorry for the confusion. All the 15 subjects included in our study are healthy controls. We have added this detailed information in Line 104 (“Fifteen healthy subjects”).

* Yet 15 subjects is very small.

AR: Thanks for the suggestions. We agree with the reviewer that 15 subjects are limited, compared to those resting-state or task-based fMRI analyses using deep learning methods (Di Martino et al., 2014; Qiang et al., 2020; Yang et al., 2020; W. Zhang et al., 2019). In our study, we used fMRI dataset under naturalistic conditions, which employed multimodal and dynamic stimuli and resembled the perceptual and cognitive experiences in real life. Despite the dynamic and complex nature of NfMRI, the proposed framework can identify complex, interactive and hierarchical FBNs from NfMRI. However, to our best knowledge, there are few public NfMRI datasets available, where the most widely-used and biggest one is provided by Human Connectome Project (HCP, https://www.humanconnectome.org/hcp-protocols-ya-7t-imaging). Specifically, in the HCP 7T release, there are 184 subjects included in the NfMRI dataset, where subjects watch a video composed of many short independent movie clips from different movies during scanning. However, previous literature revealed that compared with short independent video clips, lengthy and continuous video stimuli that are imbued with a unifying narrative could be naturally engaging, driving hierarchical neural systems matched to their implicit complexity, thus eliciting more consistent engagement of heteromodal cortex (Hasson et al., , 2008; Honey et al., 2012; Sonkusare et al., 2019; Tononi et al., 1996). Thus, the NfMRI dataset in HCP is not suitable for exploring spatial and temporal hierarchy of brain function.

On the other hand, there exist many literatures adopting NfMRI data with limited number of research subjects (Nguyen et al., 2016; Y. Ren et al., 2017; Y. D. Ren et al., 2018; van der Meer et al., 2020; Wang et al., 2017). For instance, Meer et al. used NfMRI data from seventeen healthy subjects to uncover the reshaping of functional network expression and reliable brain state dynamics (van der Meer et al., 2020). Nguyen et al. employed a public NfMRI dataset including 20 healthy participants to investigate the network mechanism of dynamic emotional experiences (Nguyen et al., 2016). All these studies show the possibility and effectiveness of small-sample NfMRI data in exploring the temporal and spatial features of the brain function.

Therefore, we believe that our dataset is sufficient to identify accurate and abundant FBNs. Nonetheless, we still admit that a large number of subjects and diverse kinds of naturalistic stimuli will make our research more accurate and convincing. Therefore, in the future work, we will apply our model to datasets with larger sample size and richer types of naturalistic stimuli. According to this suggestion, we have added additional discussion in the Discussion part (Line 496-499 “In the future work, we will focus on ...”).

* What is the number of voxels?

AR: We would like to clarify that each subject in our studies has 70831 voxels. According to this suggestion, we have added clearer description of dimension of data in Line 122 (“and each row refers ...”) and Line 242 to 243 (“p represents the number of voxels ...”)

3- On metrics:

* Spatial pattern overlap rate R defined in eq. (4) does not seem to well measure the similarities between individual level and group level networks by taking the intersection in the numerator. Isn’t Pearson correlation a better measure? How about other metrics in this application?

AR: Thanks for the suggestions. The overlap rate is a widely-used metric to measure the similarity in spatial distribution between the identified FBNs and resting state network (RSNs) templates in previous studies (Cui et al., 2020; Qiang et al., 2021; Zhao et al., 2018). However, we recognize that the overlap rate cannot measure the correlation between FBNs in terms of voxel activation. Therefore, we agree with the reviewer that Pearson correlation is a better metric to measure the similarity between FBNs.

Specifically, we used the spatial correlation coefficient (SCC) to measure the similarity between networks, of which calculation formula is defined by applying Pearson correlation to spatial voxels (Gong et al., 2018). The experimental results (Figure 7) demonstrated that those FBNs that are associated with primary sensory processes, including auditory network, medial-visual network, sensorimotor network and occipital pole-visual network, exhibited higher spatial correlation coefficient (0.68, 0.64, 0.63, 0.62, respectively). In comparison, for those networks associated with higher-order cognitive processes or emotional perception, such as default mode network and executive control network, there are significant decreases in their SCC (0.55, 0.49, respectively). In addition, we also quantitively evaluated the differences in SCC values among different networks (Table 1). Overall, the conclusion obtained by SCC analyses is consistent with previous results derived by overlap rate. According to this suggestion, we have added additional descriptions on SCC metric in Materials and Methods part (Line 234-247 “The two-stage NAS-vsDBN model can ...”) and included additional results in Results part (Line 402-430 “We thus calculated the spatial correlation coefficient ...”). In addition, we have moved experimental results using overlap rate to Extended Data (Fig. 6-1 and Table 1-1).

Figure. 7. The spatial correlation coefficient (mean, minimum and maximum) between each individual-level FBN and corresponding group-level FBN. (AUD, auditory; V1, medial visual; SM, sensorimotor; V2, occipital pole visual; DA, dorsal attention; SA, salience; DMN, default mode network; EC, executive control).

TABLE 1. The p-values of two-sample t-tests on the spatial correlation coefficient among 8 representative FBNs. (AUD, auditory; V1, medial visual; SM, sensorimotor; V2, occipital pole visual; DA, dorsal attention; SA, salience; DMN, default mode network; EC, executive control)

AUD V1 SM V2 DA SA DM EC

AUD

V1 0.056

SM 0.034 1

V2 9×10-4 0.464 0.504

DA 1×10-3 0.147 0.153 0.356

SA 1×10-4 0.038 0.038 0.088 0.708

DM 6×10-8 1×10-4 1×10-4 1×10-4 0.034 0.056

EC 8×10-10 4×10-7 3×10-7 2×10-7 1×10-4 1×10-4 0.009

4- Typo:

Line 210 and 211, is it W1*W2*W3 or W3*W2*W1 to be visualized?

The same about W1*W2.

AR: Thanks for pointing out this. We apologize for this typo and have corrected it in the current version (Line 219).

Extended Response

AR: In the legend of Figure 5, the error bar indicates standard deviation rather than standard error of mean. We would like to apologize for this typo during editing process, and have corrected it in the current version (Line 616-617).

Reference

Cui, Y., Zhao, S. J., Chen, Y. W., Han, J. W., Guo, L., Xie, L., & Liu, T. M. (2020). Modeling Brain Diverse and Complex Hemodynamic Response Patterns via Deep Recurrent Autoencoder. Ieee Transactions on Cognitive and Developmental Systems, 12(4), 733-743.

Di Martino, A., Yan, C. G., Li, Q., Denio, E., Castellanos, F. X., Alaerts, K., . . . Milham, M. P. (2014). The autism brain imaging data exchange: towards a large-scale evaluation of the intrinsic brain architecture in autism. Molecular Psychiatry, 19(6), 659-667.

Dai, H. X., Ge, F. F., Li, Q., Zhang, W., Liu, T. M., & Ieee. (2020, Apr 03-07). Optimize CNN model for fMRI signal classification via adanet based neural architecture search. Paper presented at the IEEE 17th International Symposium on Biomedical Imaging (ISBI).

Dong, Q. L., Ge, F. F., Ning, Q., Zhao, Y., Lv, J. L., Huang, H., . . . Liu, T. M. (2020). Modeling Hierarchical Brain Networks via Volumetric Sparse Deep Belief Network. Ieee Transactions on Biomedical Engineering, 67(6), 1739-1748.

Ferrarini, L., Veer, I. M., Baerends, E., van Tol, M. J., Renken, R. J., van der Wee, N. J. A., . . . Milles, J. (2009). Hierarchical Functional Modularity in the Resting-State Human Brain. Human Brain Mapping, 30(7), 2220-2231.

Fischer, A., & Igel, C. (2012). An Introduction to Restricted Boltzmann Machines. Springer Berlin Heidelberg, 7441, 14-36.

Golland, Y., Bentin, S., Gelbard, H., Benjamini, Y., Heller, R., Nir, Y., . . . Malach, R. (2007). Extrinsic and intrinsic systems in the posterior cortex of the human brain revealed during natural sensory stimulation. Cerebral Cortex, 17(4), 766-777.

Gong, J. H., Liu, X. Y., Liu, T. M., Zhou, J. S., Sun, G., & Tian, J. X. (2018). Dual Temporal and Spatial Sparse Representation for Inferring Group-Wise Brain Networks From Resting-State fMRI Dataset. Ieee Transactions on Biomedical Engineering, 65(5), 1035-1048.

Hasson, U., Yang, E., Vallines, I., Heeger, D. J., & Rubin, N. (2008). A hierarchy of temporal receptive windows in human cortex. Journal of Neuroscience, 28(10), 2539-2550.

Honey, C. J., Thesen, T., Donner, T. H., Silbert, L. J., Carlson, C. E., Devinsky, O., . . . Hasson, U. (2012). Slow Cortical Dynamics and the Accumulation of Information over Long Timescales. Neuron, 76(2), 423-434.

Li, Q., Wu, X., & Liu, T. M. (2021). Differentiable neural architecture search for optimal spatial/temporal brain function network decomposition. Medical Image Analysis, 69, 14.

Nguyen, V. T., Breakspear, M., Hu, X. T., & Guo, C. C. (2016). The integration of the internal and external milieu in the insula during dynamic emotional experiences. Neuroimage, 124, 455-463.

Park, H. J., & Friston, K. J. (2013). Structural and Functional Brain Networks: From Connections to Cognition. Science, 342(6158), 579.

Qiang, N., Dong, Q. L., Ge, F. F., Liang, H. T., Ge, B., Zhang, S., . . . Liu, T. M. (2021). Deep Variational Autoencoder for Mapping Functional Brain Networks. Ieee Transactions on Cognitive and Developmental Systems, 13(4), 841-852.

Qiang, N., Dong, Q. L., Zhang, W., Ge, B., Ge, F. F., Liang, H. T., . . . Liu, T. M. (2020). Modeling task-based fMRI data via deep belief network with neural architecture search. Computerized Medical Imaging and Graphics, 83, 12.

Ren, Y., Nguyen, V. T., Guo, L., & Guo, C. C. (2017). Inter-subject Functional Correlation Reveal a Hierarchical Organization of Extrinsic and Intrinsic Systems in the Brain. Scientific Reports, 7, 12.

Ren, Y. D., Nguyen, V. T., Sonkusare, S., Lv, J. L., Pang, T. J., Guo, L., . . . Guo, C. C. (2018). Effective connectivity of the anterior hippocampus predicts recollection confidence during natural memory retrieval. Nature Communications, 9, 10.

Ren, Y., Xu, S., Tao, Z., Song, L., He, X. (2021a). Hierarchical spatio-temporal modeling of naturalistic functional magnetic resonance imaging signals via two-stage deep belief network with neural architecture search. Frontiers in Neuroscience, 15, 13.

Ren, Y., Tao, Z., Zhang, W., Liu, T. (2021b). Modeling hierarchical spatial and temporal patterns of naturalistic fMRI volume via volumetric deep belief network with neural architecture search. Paper presented at the 2021 IEEE 18th International Symposium on Biomedical Imaging.

Schmithorst, V. J., & Holland, S. K. (2004). Comparison of three methods for generating group statistical inferences from independent component analysis of functional magnetic resonance imaging data. Journal of Magnetic Resonance Imaging, 19(3), 365-368.

Shi, Y., & Eberhart, R. C. (1998). Parameter selection in particle swarm optimization. Paper presented at the International Conference on Evolutionary Programming, Heidelberg.

Shi, Y., & Eberhart, R. C. (1999). Empirical study of particle swarm optimization. Paper presented at the 1999 Congress on Evolutionary Computation-CEC99, Washington, DC, USA.

Sonkusare, S., Breakspear, M., & Guo, C. (2019). Naturalistic Stimuli in Neuroscience: Critically Acclaimed. Trends in Cognitive Sciences, 23(8), 699-714.

Tononi, G., Sporns, O., & Edelman, G. M. (1996). A complexity measure for selective matching of signals by the brain. Proceedings of the National Academy of Sciences of the United States of America, 93(8), 3422-3427.

van der Meer, J. N., Breakspear, M., Chang, L. J., Sonkusare, S., & Cocchi, L. (2020). Movie viewing elicits rich and reliable brain state dynamics. Nature Communications, 11(1), 14.

Wang, J. H., Ren, Y. D., Hu, X. T., Nguyen, V. T., Guo, L., Han, J. W., & Guo, C. C. (2017). Test-Retest Reliability of Functional Connectivity Networks During Naturalistic fMRI Paradigms. Human Brain Mapping, 38(4), 2226-2241.

Yang, X., Schrader, P. T., & Zhang, N. (2020). A Deep Neural Network Study of the ABIDE Repository on Autism Spectrum Classification. International Journal of Advanced Computer Science and Applications, 11(4), 1-6.

Zhang, W., Zhao, L., Li, Q., Zhao, S. J., Dong, Q. L., Jiang, X., . . . Liu, T. M. (2019, Oct 13-17). Identify Hierarchical Structures from Task-Based fMRI Data via Hybrid Spatiotemporal Neural Architecture Search Net. Paper presented at the 10th International Workshop on Machine Learning in Medical Imaging (MLMI) / 22nd International Conference on Medical Image Computing and Computer-Assisted Intervention (MICCAI).

Zhang, Y., Hu, X. T., He, C. L., Wang, X. N., Ren, Y. D., Liu, H., . . . Ieee. (2019, Apr 08-11). A TWO-STAGE DBN-BASED METHOD TO EXPLORING FUNCTIONAL BRAIN NETWORKS IN NATURALISTIC PARADIGM FMRI. Paper presented at the 16th IEEE International Symposium on Biomedical Imaging (ISBI).

Zhao, Y., Dong, Q. L., Zhang, S., Zhang, W., Chen, H. B., Jiang, X., . . . Liu, T. M. (2018). Automatic Recognition of fMRI-Derived Functional Networks Using 3-D Convolutional Neural Networks. Ieee Transactions on Biomedical Engineering, 65(9), 1975-1984.

## References

[B1] Allen EA, Damaraju E, Plis SM, Erhardt EB, Eichele T, Calhoun VD (2014) Tracking whole brain connectivity dynamics in the resting state. Cereb Cortex 24:663–676. 10.1093/cercor/bhs352 23146964 PMC3920766

[B2] Beckmann CF, Jenkinson M, Smith SM (2003) General multilevel linear modeling for group analysis in fMRI. Neuroimage 20:1052–1063. 10.1016/S1053-8119(03)00435-X 14568475

[B3] Calhoun VD, Miller R, Pearlson G, Adalı T (2014) The chronnectome: time-varying connectivity networks as the next frontier in fMRI data discovery. Neuron 84:262–274. 10.1016/j.neuron.2014.10.015 25374354 PMC4372723

[B4] Catalino MP, Yao S, Green DL, Laws ER, Golby AJ, Tie Y (2020) Mapping cognitive and emotional networks in neurosurgical patients using resting-state functional magnetic resonance imaging. Neurosurg Focus 48:E9. 10.3171/2019.11.FOCUS19773 PMC7712886 32006946

[B5] Cui Y, Zhao SJ, Wang H, Xie L, Chen YW, Han JW, Guo L, Zhou F, Liu TM (2019) Identifying brain networks at multiple time scales via deep recurrent neural network. IEEE J Biomed Health Inform 23:2515–2525. 10.1109/JBHI.2018.2882885 30475739 PMC6914656

[B6] Dai HX, Ge FF, Li Q, Zhang W, Liu TM (2020) Optimize CNN model for fMRI signal classification via adanet based neural architecture search. Paper presented at the IEEE 17th International Symposium on Biomedical Imaging (ISBI), Apr 03-07. lowa, IA:IEEE.

[B7] Damascelli M, Woodward TS, Sanford N, Zahid HB, Lim R, Scott A, Kramer JK (2021) Multiple functional brain networks related to pain perception revealed by fMRI. Neuroinformatics. Advance online publication. Retrieved Jun 8, 2021. doi: 10.1007/s12021-021-09527-6. 10.1007/s12021-021-09527-6 PMC9537130 34101115

[B8] Ferrarini L, Veer IM, Baerends E, van Tol MJ, Renken RJ, van der Wee NJA, Veltman DJ, Aleman A, Zitman FG, Penninx B, van Buchem MA, Reiber JHC, Rombouts S, Milles J (2009) Hierarchical functional modularity in the resting-state human brain. Hum Brain Mapp 30:2220–2231. 10.1002/hbm.20663 18830955 PMC6871119

[B9] Fischer A, Igel C (2012) An introduction to restricted Boltzmann machines. In: Progress in pattern recognition, image analysis, computer vision, and applications. CIARP 2012. Lecture notes in computer science, Vol 7441, 14–36. Berlin; Heidelberg: Springer.

[B10] Golland Y, Bentin S, Gelbard H, Benjamini Y, Heller R, Nir Y, Hasson U, Malach R (2007) Extrinsic and intrinsic systems in the posterior cortex of the human brain revealed during natural sensory stimulation. Cereb Cortex 17:766–777. 10.1093/cercor/bhk030 16699080

[B11] Hasson U, Nir Y, Levy I, Fuhrmann G, Malach R (2004) Intersubject synchronization of cortical activity during natural vision. Science 303:1634–1640. 15016991 10.1126/science.1089506

[B12] Hasson U, Malach R, Heeger DJ (2010) Reliability of cortical activity during natural stimulation. Trends Cogn Sci 14:40–48. 10.1016/j.tics.2009.10.011 20004608 PMC2818432

[B13] Hinton GE, Osindero S, Teh YW (2006) A fast learning algorithm for deep belief nets. Neural Comput 18:1527–1554. 10.1162/neco.2006.18.7.1527 16764513

[B14] Hu XT, Huang H, Peng B, Han JW, Liu N, Lv JL, Guo L, Guo C, Liu TM (2018) Latent source mining in fMRI via restricted Boltzmann machine. Hum Brain Mapp 39:2368–2380. 10.1002/hbm.24005 29457314 PMC6866484

[B15] Huang H, Hu XT, Zhao Y, Makkie M, Dong QL, Zhao SJ, Guo L, Liu TM (2018) Modeling task fMRI data via deep convolutional autoencoder. IEEE Trans Med Imaging 37:1551–1561. 10.1109/TMI.2017.2715285 28641247

[B16] Huang ZA, Zhu ZX, Yau CH, Tan KC (2021) Identifying autism spectrum disorder from resting-state fMRI using deep belief network. IEEE Trans Neural Netw Learn Syst 32:2847–2861. 10.1109/TNNLS.2020.3007943 32692687

[B17] Kennedy J, Eberhart R (1995) Particle swarm optimization. Paper presented at the ICNN95-International Conference on Neural Networks, Nov 27-Dec 01. Perth, WA, Australia: IEEE.

[B18] Kober H, Lacadie CM, Wexler BE, Malison RT, Sinha R, Potenza MN (2016) Brain activity during cocaine craving and gambling urges: an fMRI Study. Neuropsychopharmacology 41:628–637. 10.1038/npp.2015.193 26119472 PMC5130138

[B19] Li Q, Wu X, Liu TM (2021) Differentiable neural architecture search for optimal spatial/temporal brain function network decomposition. Med Image Anal 69:101974. 10.1016/j.media.2021.101974 33588118

[B20] Li Q, Zhang W, Zhao L, Wu X, Liu TM (2022) Evolutional neural architecture search for optimization of spatiotemporal brain network decomposition. IEEE Trans Biomed Eng 69:624–634. 34357861 10.1109/TBME.2021.3102466

[B21] Nastase SA, Gazzola V, Hasson U, Keysers C (2019) Measuring shared responses across subjects using intersubject correlation. Soc Cogn Affect Neurosci 14:669–687. 10.1093/scan/nsz037 PMC6688448 31099394

[B22] Pham H, Guan MY, Zoph B, Le QV, Dean J (2018) Efficient neural architecture search via parameter sharing. Paper presented at the 35th International Conference on Machine Learning (ICML), Jul 10-15. Stockholm, SWEDEN: IMLS.

[B23] Qiang N, Dong QL, Zhang W, Ge B, Ge FF, Liang HT, Sun YF, Gao J, Liu TM (2020) Modeling task-based fMRI data via deep belief network with neural architecture search. Comput Med Imaging Graph 83:101747. 10.1016/j.compmedimag.2020.101747 32593949 PMC7412935

[B24] Real E, Aggarwal A, Huang YP, Le QV (2019) Regularized evolution for image classifier architecture search. Paper presented at the 33rd AAAI Conference on Artificial Intelligence / 31st Innovative Applications of Artificial Intelligence Conference / 9th AAAI Symposium on Educational Advances in Artificial Intelligence, Jan 27-Feb 01. Honolulu, HI: AAAI.

[B25] Ren Y, Fang J, Lv JL, Hu XT, Guo CC, Guo L, Xu JS, Potenza MN, Liu T (2017a) Assessing the effects of cocaine dependence and pathological gambling using group-wise sparse representation of natural stimulus FMRI data. Brain Imaging Behav 11:1179–1191. 10.1007/s11682-016-9596-4 27704410 PMC5378673

[B26] Ren Y, Nguyen VT, Guo L, Guo CC (2017b) Inter-subject functional correlation reveal a hierarchical organization of extrinsic and intrinsic systems in the brain. Sci Rep 7:10876. 10.1038/s41598-017-11324-8 28883508 PMC5589774

[B27] Ren Y, Xu S, Tao Z, Song L, He X (2021a) Hierarchical spatio-temporal modeling of naturalistic functional magnetic resonance imaging signals via two-stage deep belief network with neural architecture search. Front Neurosci 15:794955. 10.3389/fnins.2021.794955 34955738 PMC8692564

[B28] Ren Y, Tao Z, Zhang W, Liu T (2021b) Modeling hierarchical spatial and temporal patterns of naturalistic fMRI volume via volumetric deep belief network with neural architecture search. Paper presented at the 2021 IEEE 18th International Symposium on Biomedical Imaging, Apr 13-16. Nice, FRANCE: IEEE.

[B29] Shi Y, Eberhart RC (1998) Parameter selection in particle swarm optimization. Paper presented at the International Conference on Evolutionary Programming, May 4-9. Anchorage, AK: IEEE.

[B30] Salman MS, Du YH, Lin DD, Fu ZN, Fedorov A, Damaraju E, Sui J, Chen JY, Mayer AR, Posse S, Mathalon DH, Ford JM, Van Erp T, Calhoun VD (2019) Group ICA for identifying biomarkers in schizophrenia: ‘adaptive’ networks via spatially constrained ICA show more sensitivity to group differences than spatio-temporal regression. Neuroimage Clin 22:101747. 30921608 10.1016/j.nicl.2019.101747 PMC6438914

[B31] Schmithorst VJ, Holland SK (2004) Comparison of three methods for generating group statistical inferences from independent component analysis of functional magnetic resonance imaging data. J Magn Reson Imaging 19:365–368. 10.1002/jmri.20009 14994306 PMC2265794

[B32] Sonkusare S, Breakspear M, Guo C (2019) Naturalistic stimuli in neuroscience: critically acclaimed. Trends Cogn Sci 23:699–714. 31257145 10.1016/j.tics.2019.05.004

[B33] Vanderwal T, Kelly C, Eilbott J, Mayes LC, Castellanos FX (2015) Inscapes: a movie paradigm to improve compliance in functional magnetic resonance imaging. Neuroimage 122:222–232. 10.1016/j.neuroimage.2015.07.069 26241683 PMC4618190

[B34] Zhang W, Zhao L, Li Q, Zhao SJ, Dong QL, Jiang X, Zhang T, Liu TM (2019) Identify hierarchical structures from task-based fMRI data via hybrid spatiotemporal neural architecture search net. Paper presented at the 10th International Workshop on Machine Learning in Medical Imaging (MLMI)/22nd International Conference on Medical Image Computing and Computer-Assisted Intervention, Oct 13-17. Shenzhen, PEOPLES R CHINA: MICCAI.

[B35] Zhang Y, Hu XT, He CL, Wang XN, Ren YD, Liu H, Wang LT, Guo L, Liu TM (2019) A two-stage DBN-based method to exploring functional brain networks in naturalistic paradigm fMRI. Paper presented at the 16th IEEE International Symposium on Biomedical Imaging (ISBI), Apr 08-11. Venice, ITALY: IEEE.

