# Supplementary figures and images for: Hierarchical Individual Naturalistic Functional Brain Networks with Group Consistency Uncovered by a Two-Stage NAS-Volumetric Sparse DBN Framework
Source: eNeuro. 2022 Sep 7;9(5):ENEURO.0200-22.2022. doi: 10.1523/ENEURO.0200-22.2022 (PMC9463984; doi:10.1523/ENEURO.0200-22.2022)

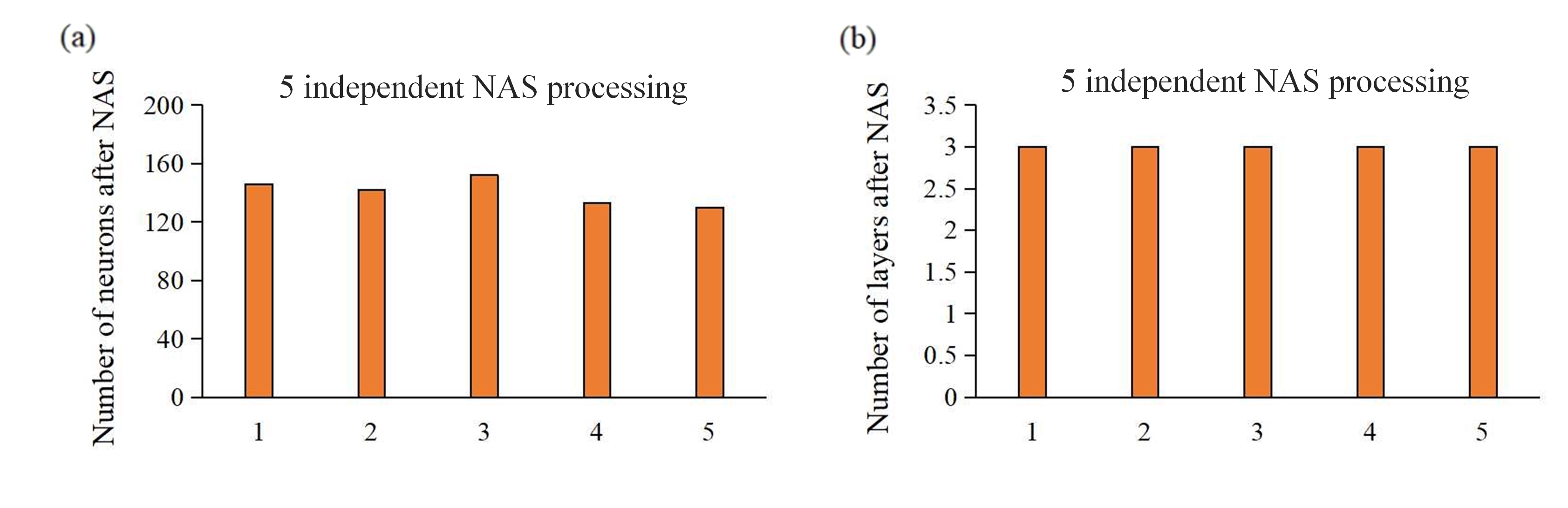

Supplement: Extended Data Figure 2-1 — Results of five independent NAS processes using linearly decreasing weight. a, Number of neurons after NAS. b, Number of layers after NAS. Download Figure 2-1, TIF file. [file enu-eN-MNT-0200-22-s03.tif]

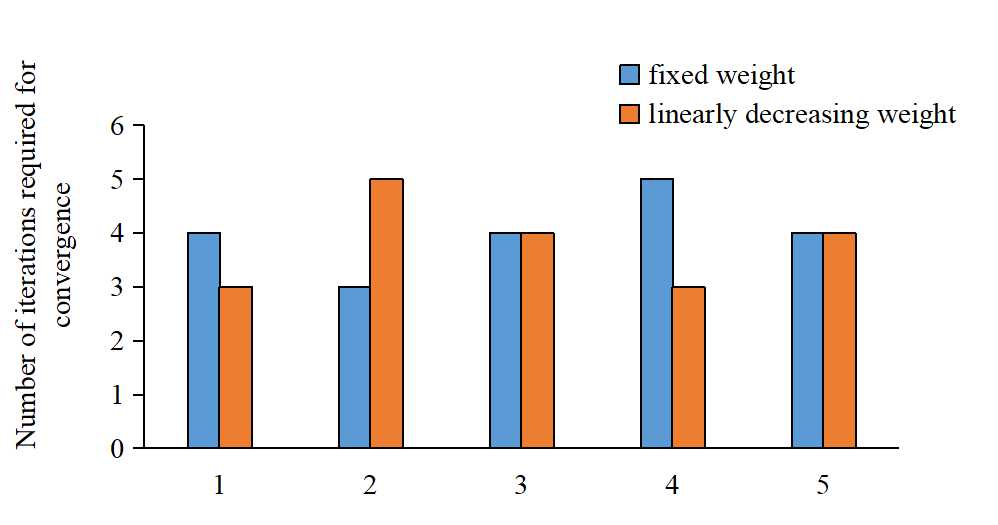

Supplement: Extended Data Figure 2-2 — Comparison of convergence speed between fixed weight and linearly decreasing weight. Download Figure 2-2, TIF file. [file enu-eN-MNT-0200-22-s04.tif]

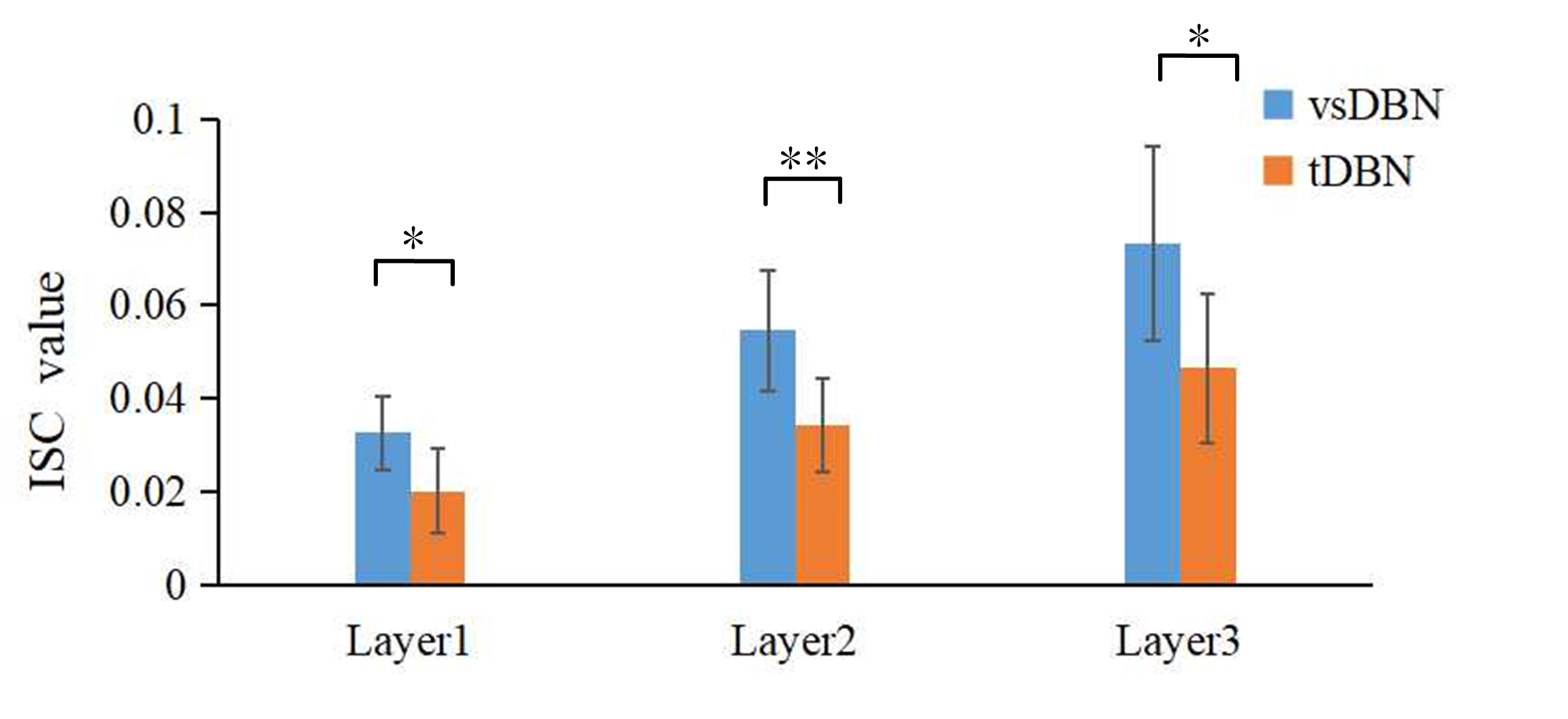

Supplement: Extended Data Figure 5-1 — Comparison of group-level ISC values between two-stage tDBN model and two-stage NAS-vsDBN model. Error bar indicates SD. The statistical test was conducted by two-sample t test, where * represents FDR-corrected p < 1 × 1 × 10−3 and ** represents p < 1 × 1 × 10−4. Download Figure 5-1, TIF file. [file enu-eN-MNT-0200-22-s05.tif]

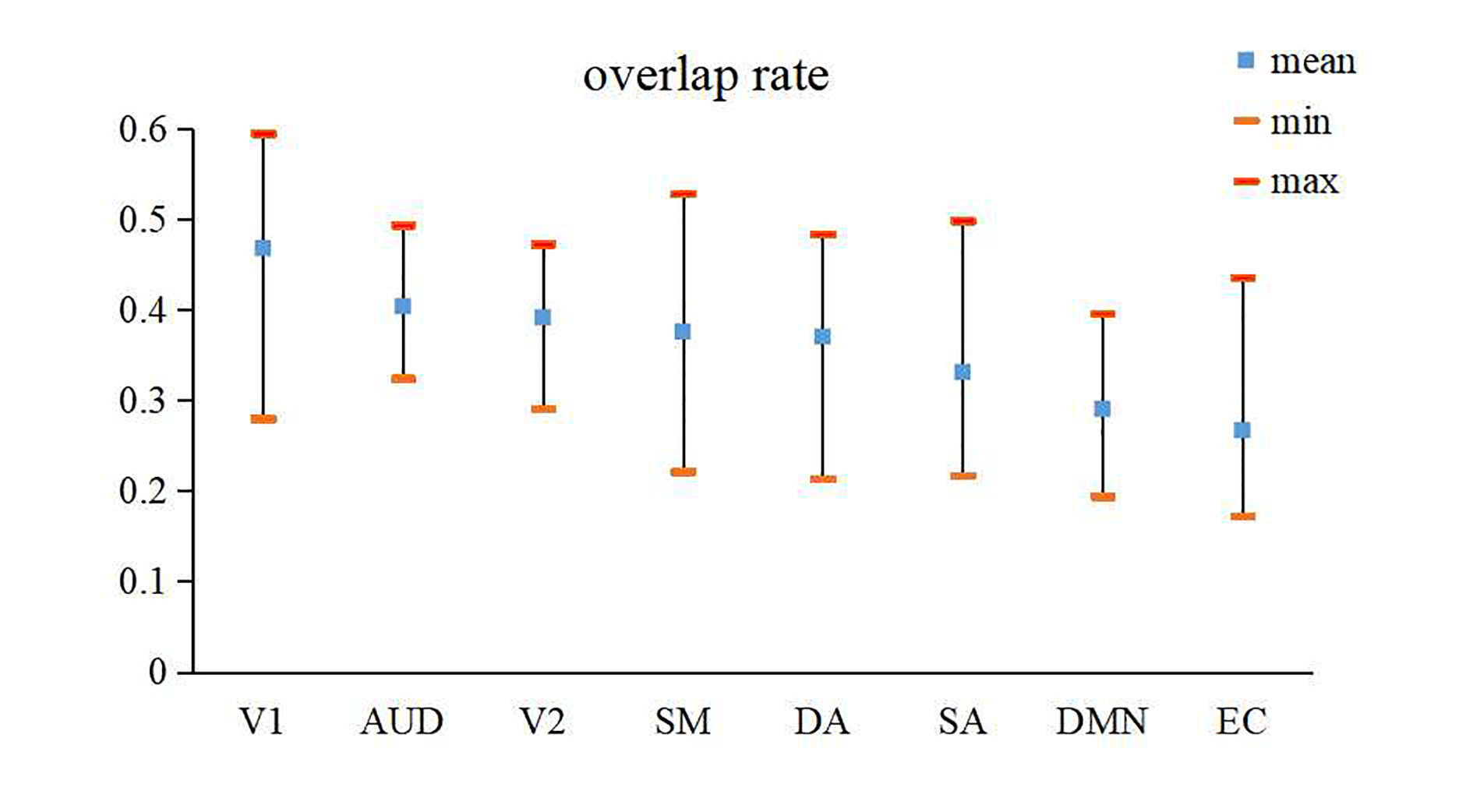

Supplement: Extended Data Figure 6-1 — The overlap rate (mean, minimum, and maximum) between each individual-level FBN and corresponding group-level FBN (V1, medial visual; AUD, auditory; V2, occipital pole visual; SM, sensorimotor; DA, dorsal attention; SA, salience; DMN, default mode network; EC, executive control). Download Figure 6-1, TIF file. [file enu-eN-MNT-0200-22-s06.tif]

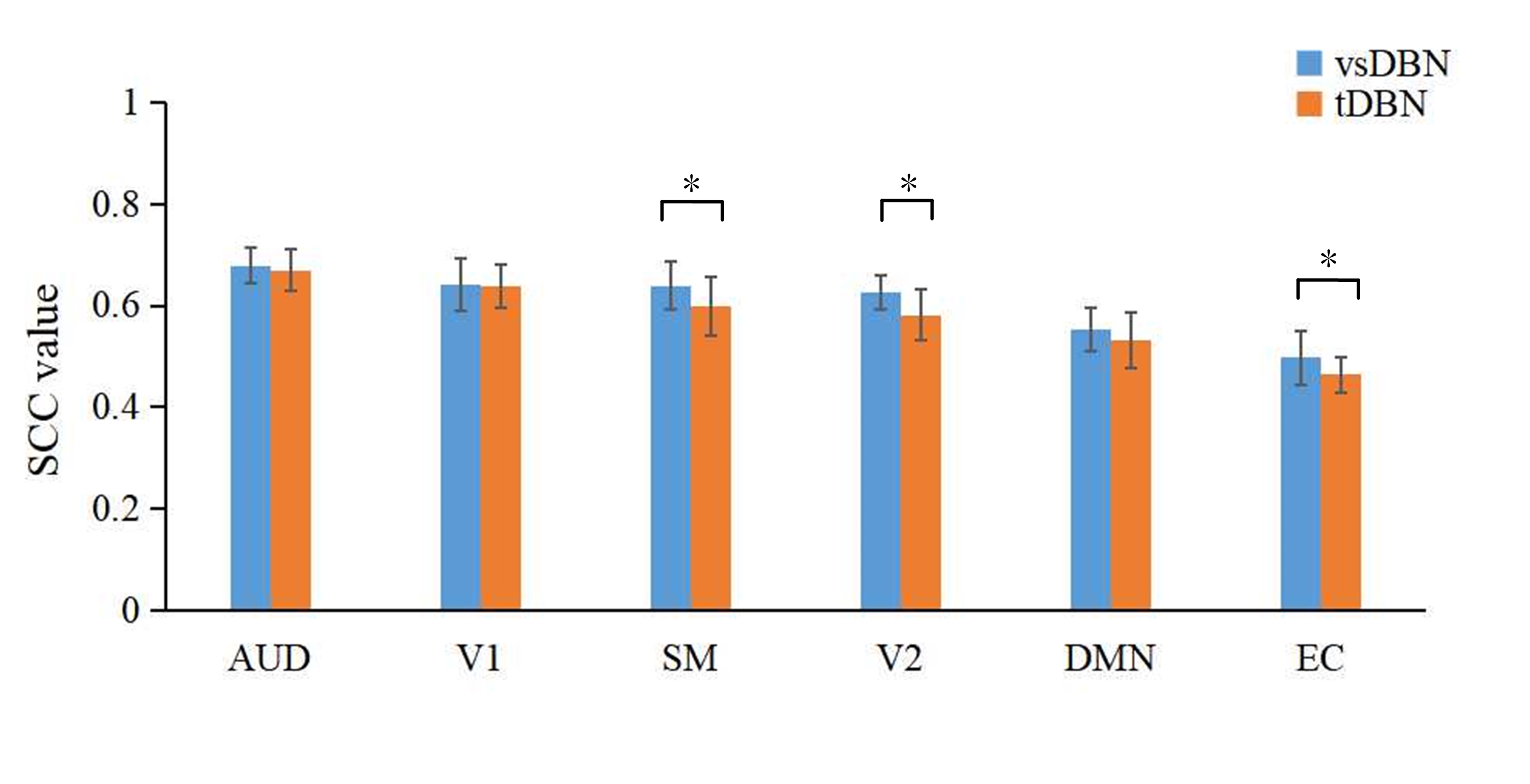

Supplement: Extended Data Figure 6-2 — Comparison of the SCC between two-stage tDBN model and two-stage NAS-vsDBN model. Error bar indicates SD. The statistical test was conducted by two-sample t test, where * represents p < 1 × 1 × 10−2 (AUD, auditory; V1, medial visual; SM, sensorimotor; V2, occipital pole visual; DMN, default mode network; EC, executive control). Download Figure 6-2, TIF file. [file enu-eN-MNT-0200-22-s07.tif]

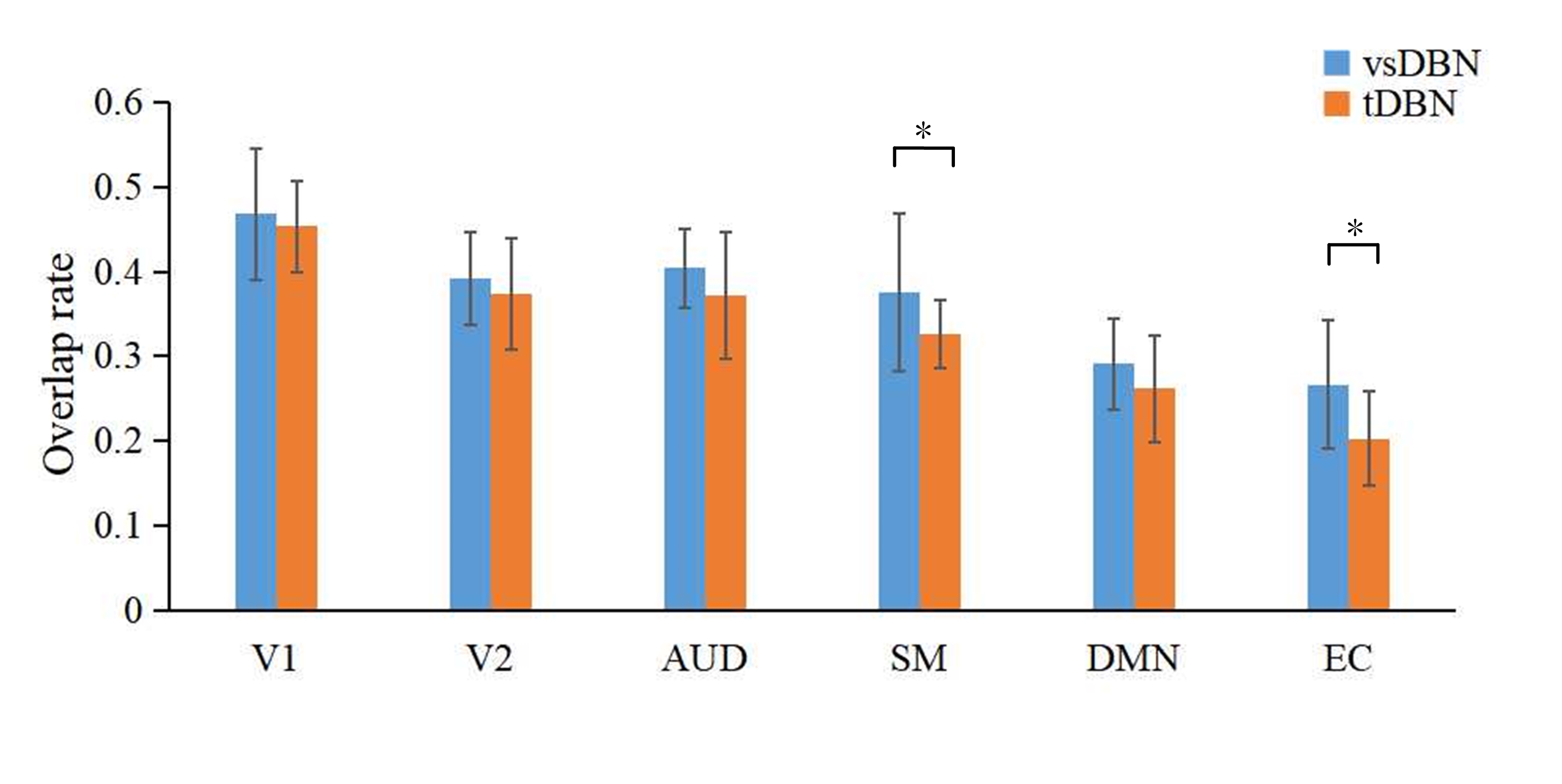

Supplement: Extended Data Figure 6-3 — Comparison of the overlap rate between two-stage tDBN model and two-stage NAS-vsDBN model. Error bar indicates SD. The statistical test was conducted by two-sample t test, where * represents p < 1 × 1 × 10−2 (V1, medial visual; V2, occipital pole visual; AUD, auditory; SM, sensorimotor; DMN, default mode network; EC, executive control). Download Figure 6-3, TIF file. [file enu-eN-MNT-0200-22-s08.tif]
