# Supplementary material for: Hierarchical Individual Naturalistic Functional Brain Networks with Group Consistency Uncovered by a Two-Stage NAS-Volumetric Sparse DBN Framework
Source: eNeuro. 2022 Sep 7;9(5):ENEURO.0200-22.2022. doi: 10.1523/ENEURO.0200-22.2022 (PMC9463984; doi:10.1523/ENEURO.0200-22.2022)
Supplement: Extended Data Table 1-1 — The p-values of two-sample t tests on the overlap rates among 8 representative FBNs (AUD, auditory; V1, medial visual; SM, sensorimotor; V2, occipital pole visual; DA, dorsal attention; SA, salience; DMN, default mode network; EC, executive control). Download Table 1-1, DOC file. [file enu-eN-MNT-0200-22-s10.doc]

TABLE 1-1. The p-values of two-sample t-tests on the overlap rates among 8 representative FBNs. (V1, medial visual; AUD, auditory; V2, occipital pole visual; SM, sensorimotor; DA, dorsal attention; SA, salience; DMN, default mode network; EC, executive control)

|  | V1 | AUD | V2 | SM | DA | SA | DM | EC |
| --- | --- | --- | --- | --- | --- | --- | --- | --- |
| V1 |  |  |  |  |  |  |  |  |
| AUD | 0.051 |  |  |  |  |  |  |  |
| V2 | 0.027 | 0.650 |  |  |  |  |  |  |
| SM | 0.028 | 0.449 | 0.701 |  |  |  |  |  |
| DA | 0.013 | 0.273 | 0.515 | 0.986 |  |  |  |  |
| SA | 0.001 | 0.021 | 0.061 | 0.296 | 0.303 |  |  |  |
| DM | 1×10^-5^ | 1×10^-5^ | 1×10^-4^ | 0.015 | 0.009 | 0.227 |  |  |
| EC | 1×10^-5^ | 1×10^-5^ | 1×10^-4^ | 0.007 | 0.004 | 0.072 | 0.449 |  |
